# Artificial Intelligence for Evidence Synthesis of Emerging Biologics to Improve Skeletal Health in Osteogenesis Imperfecta: Systematic Review and Meta-Analysis

**DOI:** 10.2196/85840

**Published:** 2026-07-10

**Authors:** Chengfei Li, Zonglin Dai, Wing Chung Tang, Zesen Gao, Vivien Kin Yi Chan, Mariana Ramirez-Posada, Jiyeong Kim, Eleni Linos, CL Cheung, Ian Chi Kei Wong, Dong Dong, Michael To, Dawn Craig, Xue Li

**Affiliations:** 1 Centre for Safe Medication Practice and Research, Department of Pharmacology and Pharmacy, Li Ka Shing Faculty of Medicine, The University of Hong Kong, Hong Kong SAR, China (Hong Kong); 2 Department of Medicine, School of Clinical Medicine, Li Ka Shing Faculty of Medicine, The University of Hong Kong, PB306, Professorial Block, Queen Mary Hospital, 102 Pok Fu Lam Road, Hong Kong SAR, China (Hong Kong), 852 2255 3319; 3 The University of Hong Kong–Shenzhen Hospital, Shenzhen, China; 4 The University of Hong Kong Libraries, The University of Hong Kong, Hong Kong SAR, China (Hong Kong); 5 Department of Dermatology, Stanford School of Medicine, Stanford, CA, United States; 6 Stanford Center for Digital Health, Department of Medicine, Stanford University, Stanford, CA, United States; 7 Department of Pharmacology and Pharmacy, Li Ka Shing Faculty of Medicine, The University of Hong Kong, Hong Kong, China (Hong Kong); 8 School of Pharmacy, Aston University, Birmingham, United Kingdom; 9 Macau University of Science and Technology, Macau Special Administrative Region, China; 10 The Jockey Club School of Public Health and Primary Care (JCSPHPC),The Chinese University of Hong Kong, New Territories, Hong Kong SAR, China (Hong Kong); 11 Department of Orthopaedics and Traumatology, Li Ka Shing Faculty of Medicine, The University of Hong Kong, Hong Kong, China (Hong Kong); 12 Shenzhen Clinical Research Center for Rare Diseases, Shenzhen, China; 13 Population Health Sciences Institute, Faculty of Medical Sciences, Newcastle University, Newcastle upon Tyne, United Kingdom

**Keywords:** artificial intelligence, ChatGPT, evidence synthesis, biologics, osteogenesis imperfecta

## Abstract

**Background:**

Osteogenesis imperfecta (OI) is a rare genetic disorder characterized by bone fragility and recurrent fractures. Emerging biologics demonstrate promise by targeting bone-remodeling pathways, yet evidence for their efficacy and safety remains fragmented and heterogeneous, and no prior systematic review in OI has incorporated artificial intelligence (AI) to synthesize it.

**Objective:**

This study aims to systematically evaluate the efficacy and safety of novel biologics in patients with OI using an AI-assisted workflow for evidence synthesis.

**Methods:**

We conducted a systematic review and meta-analysis of interventional trials of denosumab, setrusumab, teriparatide, romosozumab, and fresolimumab. Data were retrieved from PubMed, Web of Science, Embase, ScienceDirect, the Cochrane Library, and ClinicalTrials.gov up to December 1, 2025. Eligible studies enrolled individuals with OI, reported areal bone mineral density (aBMD) and/or fractures, and were randomized, nonrandomized, or single-arm studies; case series were excluded. As a methodological feature, GPT-4o was integrated into the workflow to perform a parallel 2-stage screening (title/abstract and full text) and to assist with risk of bias assessment using an adapted Cochrane RoB 2 tool. The primary outcome, percentage change in aBMD, was synthesized using a random-effects meta-analysis. GPT-4o was benchmarked against human reviewers using sensitivity, specificity, and weighted Cohen κ.

**Results:**

Thirteen trials (n=684) were systematically reviewed, of which 10 (n=333) contributed to meta-analyses. In children, denosumab produced the greatest 12-month increase in lumbar spine aBMD (25.49%, 95% CI 17.14%‐33.84%). In adults, setrusumab at 12 months yielded the highest improvement (9.38%, 95% CI 6.5%‐12.26%). Across trials, no biologic significantly reduced fracture incidence compared to bisphosphonates. Safety profiles varied: denosumab was associated with a high risk of hypercalcemia in children (30.95%), whereas setrusumab had no treatment-related serious adverse events. AI achieved high sensitivity in abstract (97.4%) and full-text (88.9%) screening, and reduced total screening time by over 95%. Although there was substantial agreement with humans in the quality assessment (Cohen κ=0.778, 95% CI 0.710‐0.846), the model exhibited optimism and positional biases due to reliance on probabilistic language patterns rather than structured clinical reasoning.

**Conclusions:**

This review is the first to synthesize and quantitatively compare skeletal outcomes across multiple biologics in OI with an AI-assisted review workflow. Denosumab and setrusumab demonstrate promising efficacy in improving lumbar spine aBMD across ages, although current evidence does not support superior fracture reduction over bisphosphonates. GPT-4o can substantially accelerate evidence synthesis but should be deployed with explicit human oversight in tasks requiring contextual understanding and clinical reasoning. These findings should be interpreted cautiously given the small and heterogeneous trial base. Taken together, our workflow presented how evidence synthesis may be scaled and operationalized in real-world rare disease research.

## Introduction

Osteogenesis imperfecta (OI) is a rare genetic connective tissue disorder with an estimated incidence of 1 in 15,000 to 20,000 live births [1]. It is characterized not only by bone fragility, recurrent fractures, and bone deformities, but also by growth impairment, chronic bone pain, and hearing loss, all of which contribute to lifelong disability and reduced health-related quality of life [2]. OI is primarily caused by mutations affecting type I collagen, the principal structural protein in bone [3], resulting in an abnormal collagen matrix and structurally fragile bone [4]. To date, more than 22 subtypes have been identified, with type I OI being the most common and clinically mild, whereas other subtypes (eg, types II-IV) are more severe [5,6].

Current therapeutic strategies for OI remain palliative, focusing only on symptomatic relief. Bisphosphonates (BPs), such as alendronate, zoledronate, and neridronate, are small-molecule antiresorptive agents that increase bone mass by nonselectively binding to bone surface hydroxyapatite and inhibiting osteoclast-mediated bone resorption. For the past two decades, they have been widely recommended and routinely used as first-line therapy for OI, particularly in children with moderate-to-severe disease. However, several systematic reviews of randomized controlled trials (RCTs) have shown that they are less effective in adults or patients with severe phenotypes (eg, types II-IV) [7-9] and are associated with common side effects such as upper gastrointestinal irritation and acute-phase reactions [10].

In parallel, emerging biologics—such as denosumab (RANKL inhibition) [11], setrusumab and romosozumab (sclerostin neutralization) [5,12], teriparatide (parathyroid hormone activation) [13], and fresolimumab (TGF-β inhibition) [14]—provide more targeted mechanisms of action by modulating specific molecular pathways in bone remodeling. These biologics offer potential for improved efficacy and fewer off-target side effects compared to BPs. But their clinical value remains uncertain: most data come from early-phase single-arm studies or small randomized trials, with heterogeneous follow-up durations and inconsistent findings for functional status and fracture outcomes. Existing syntheses are limited by narrow drug scope, age-restricted samples, and variability in study design. Few have quantitatively pooled safety and efficacy data across biologic classes, and none, to our knowledge, have systematically evaluated biologics in both adults and children with OI using meta-analytic methods. This evolving yet fragmented evidence base underscores the need for an up-to-date synthesis to inform clinical decision-making.

However, conducting such comprehensive reviews in OI is challenging. Heterogeneous study designs and inconsistent outcomes make manual screening and appraisal labor-intensive and prone to cognitive fatigue [15,16]. Advances in large language models (LLMs) offer promising avenues to reduce human workload by automating parts of the systematic review workflow, but current applications are limited in both task scope and methodological rigor. Prior studies have primarily focused on narrow aspects such as Boolean query optimization [17] or abstract-level screening [18]. GPT-4o has been applied to full-text analysis [19], yet artificial intelligence (AI)–driven quality appraisal was omitted. Beyond these technical limitations, existing implementations may introduce human-AI interaction biases; for example, automation bias may lead reviewers to over-trust model-generated inclusion decisions [20,21], and conversely, a conventional “human-in-the-loop” framework can reproduce human biases and further compromise the generalizability of the model [22,23]. Collectively, these limitations call for a more comprehensive evaluation of LLMs in evidence synthesis—one that goes beyond isolated tasks and combines human expertise with automation in a way that is reliable and generalizable to real-world review workflows.

Therefore, we conducted a systematic review and meta-analysis of clinical trials of biologics for OI to evaluate their efficacy and safety. To support the review process, we also implemented an LLM-assisted workflow that simulates integrated human screening and appraisal, and evaluated its agreement with consensus decisions by human reviewers.

## Methods

We followed the PRISMA (Preferred Reporting Items for Systematic Reviews and Meta-Analyses) 2020 reporting guideline and referred to the PRISMA 2020 expanded checklist for detailed explanations (Checklist 1) [24].

### Search Strategy and Eligibility Criteria

To first identify novel biologics under investigation for the treatment of OI, we horizontally scanned ScanMedicine and ClinicalTrials.gov for all phases of clinical trials registered up to December 1, 2025. Trials were excluded if they were withdrawn, suspended, or conducted solely in animal models. Only those reporting preliminary efficacy outcomes were included. Through this process, we identified 7 biologics: teriparatide, fresolimumab, setrusumab, romosozumab, blosozumab, somatropin, and denosumab.

To further evaluate the clinical efficacy of biologics, we systematically searched PubMed, Embase, ScienceDirect, Web of Science, and the Cochrane Library for human studies published up to December 1, 2025. All databases and registries were searched separately, not via a multidatabase platform. No additional trial registries (eg, WHO ICTRP) were searched. The search terms combined free-text in titles, abstracts, and keywords: (“osteogenesis imperfecta” OR “fragilitas ossium” OR “dysostosis” OR “osteopsathyrosis” OR “brittle bone disease”) AND (“teriparatide” OR “fresolimumab” OR “setrusumab” OR “BPS804” OR “romosozumab” OR “blosozumab” OR “TST002” OR “somatropin” OR “denosumab”). Because search syntax differs across interfaces, the full database-specific strategies (including field tags/modifiers) are reported in Table S1 in Multimedia Appendix 1. Additionally, ClinicalTrials.gov was searched for relevant ongoing or completed but unpublished trials by entering “osteogenesis imperfecta” in the Condition or Disease field and the investigational drug name in the Intervention/Treatment field.

We also undertook supplementary browsing of conference proceedings and pharmaceutical press releases to supplement outcome data when original trial results were not available. In addition, we hand-searched the reference lists of included trials for additional eligible articles and contacted trial investigators when necessary to obtain missing information, such as standard deviations. Forward citation searching using citation indexes was not conducted. No published search filters were applied. Searches were run once, with restriction to English-language publications and a last update on December 1, 2025, for all databases; no study-design limits were otherwise imposed. The search strategy was developed de novo, was not adapted from previous reviews, and was formally peer-reviewed by a third information specialist.

Table S1 in Multimedia Appendix 1 also reports the number of records retrieved from each database and the total number of records before and after deduplication in EndNote (Clarivate). All records were imported into EndNote 21 for automated deduplication and then exported to Microsoft Excel for additional manual removal of duplicates; therefore, post-deduplication counts are only available for the overall dataset, not for each individual database. The literature search and their reporting followed the PRISMA-S checklist (Checklist 2) [25].

### Selection Process

#### Manual Screening

Studies were eligible for inclusion if they (1) were RCTs, non-RCTs, quasi-randomized trials, crossover trials, prospective interventional open-label trials (including single-arm studies), or historical-control interventional trials; (2) reported the efficacy outcomes of biologics identified from the horizontal scanning; and (3) provided a well-established diagnosis and subtypes of OI. Studies were excluded if they (1) were observational studies, case reports, books, or reviews; (2) investigated interventions that combined the biologics of interest with BPs or other nonlisted agents which could confound treatment effects; (3) recruited patients with impaired renal function, liver disease, hypocalcemia, or other comorbidities affecting bone metabolism, except for postmenopausal osteoporosis; and (4) did not report quantified results, especially changes in areal bone mineral density (aBMD) or fracture incidence. Detailed criteria, including the general criteria used at the title/abstract screening stage and the more specific outcome-based criteria used at the full-text screening stage, are provided in Table S2 in Multimedia Appendix 1. Two reviewers independently screened titles and abstracts for eligibility and retrieved full texts where applicable. Discrepancies were resolved through discussion until a consensus was reached.

#### LLM-Assisted Screening

To evaluate the screening capabilities of LLMs, a screening tool powered by the GPT-4o model via the Azure OpenAI API was used. Inclusion and exclusion criteria were encoded into zero-shot structured prompts. To mirror manual procedures, the process was conducted in two stages: title/abstract screening and full-text screening. Before initiating full-text screening, studies without accessible full-text documents were excluded, and only available full-text PDFs were provided to the model. During the first stage, the model categorized each study into 1 of 3 levels of relevance: “yes” (included), “no” (excluded), and “maybe” (potentially included). Studies classified as “yes” or “maybe” at the initial stage were automatically advanced to full-text screening for further evaluation. The performance of the LLM-assisted tool was benchmarked against the results of manual review, using sensitivity (recall) and specificity as the primary metrics. To facilitate reproducibility, the zero-shot structured prompts for both title/abstract and full-text screening, along with the output schema, are provided in Multimedia Appendix 2.

### Data Collection and Outcomes

Two human reviewers independently performed data extraction using a standardized form, and only articles that passed human screening were included. Extracted data included (1) innovative medicine; (2) first author and publication year; (3) study design; (4) follow-up period; (5) types of OI; (6) methods of diagnosis; (7) participants (gender and age); (8) sample size and intervention (per group); (9) primary efficacy outcomes (changes in bone mineral density [BMD], fracture incidence, functional or pain scores, and other prespecified clinical endpoints); and (10) secondary safety outcomes (adverse events, serious adverse events, and mortality). If the required data were missing, attempts were made to contact the authors to request the additional information for clarification.

### Risk of Bias Assessment

#### Manual Assessment

The quality of clinical trials was assessed using the revised Cochrane risk-of-bias tool for randomized trials (RoB 2) [26]. The tool evaluates risk of bias across 5 domains through 22 signaling questions, and each item was rated as yes (Y), probably yes (PY), probably no (PN), no (N), no information (NI), or not available (NA). For single-arm trials without control groups, specific RoB 2 items were modified by refining comparator-related domains and adjusting criteria for randomization and allocation. Two reviewers independently conducted the assessment, with disagreements resolved in consensus. Detailed risk of bias assessments, including the modified criteria for single-arm trials, are presented in Section S3 in Multimedia Appendix 1.

#### LLM-Based Assessment

An LLM-assisted approach was used to streamline the risk of bias assessment. Customized RoB 2 prompts, aligned with original guidelines, were integrated into the GPT-4o API to systematically analyze full-text PDFs selected by humans and generate standardized judgments for all signaling questions. Details of the RoB 2 prompts, including adapted signaling questions, response options, and parsing instructions, are provided in Multimedia Appendix 3.

To evaluate the interrater reliability between human raters and the GPT-4o, we calculated weighted Cohen κ coefficients for each paper, applying 5 ordinal rating categories [27]. Kappa values were computed in IBM SPSS Statistics, with linear weights of 1, 0.75, 0.5, 0.25, and 0 assigned to Y, PY, NI, PN, and N, respectively. NA, coded as 999 in the dataset, were treated as missing values in the analysis. The overall weighted κ coefficient was derived by aggregating article-level κ values using the inverse-variance weighting method. Consistent with widely accepted benchmarks, a global weighted κ coefficient of 0.6 or higher was classified as substantial agreement, reflecting high interrater consistency [27].

### Data Synthesis

For drugs with at least two trials, meta-analyses of efficacy outcomes were performed using the meta package in R (version 4.4.3), applying a random-effects model with the Hartung-Knapp-Sidik-Jonkman (HKSJ) method to account for between-study heterogeneity [28]. The primary efficacy outcome was the BMD *z* score, percentage change in BMD from baseline, and fracture incidence. For the main analyses, we conducted single-arm pre-post meta-analyses by pooling only the innovative-treatment arms from each eligible trial, irrespective of whether the original study was randomized or nonrandomized. Comparative effectiveness, when available, is reported separately in Table S4 in Multimedia Appendix 1 [14,29-39]. Each clinical trial contributed only a single effect estimate to each pooled analysis, thereby avoiding any double-counting of trials.

In accordance with Cochrane guidelines, publication bias was not formally assessed due to the limited number of studies (<10 per analysis), thus precluding the use of the Egger regression or funnel plot asymmetry tests. To address age-related heterogeneity, we first performed a primary meta-analysis at the all-age level, including all eligible trials, then conducted subgroup analyses stratified by age group (adults vs children). Additionally, to examine the impact of original study design, we conducted sensitivity analyses stratified by study design, pooling (1) intervention arms from randomized trials separately from (2) nonrandomized single-arm/self-controlled studies; results are presented in Figure S1 (a-e) in Multimedia Appendix 1. Results for drugs that were not eligible for pooling with fewer than two trials or only qualitative, nonpooled efficacy data are narratively summarized in Section S4a in Multimedia Appendix 1.

## Results

### Study Selection: GPT-4o–Assisted Screening vs Human Reviewers

**Figure 1.**
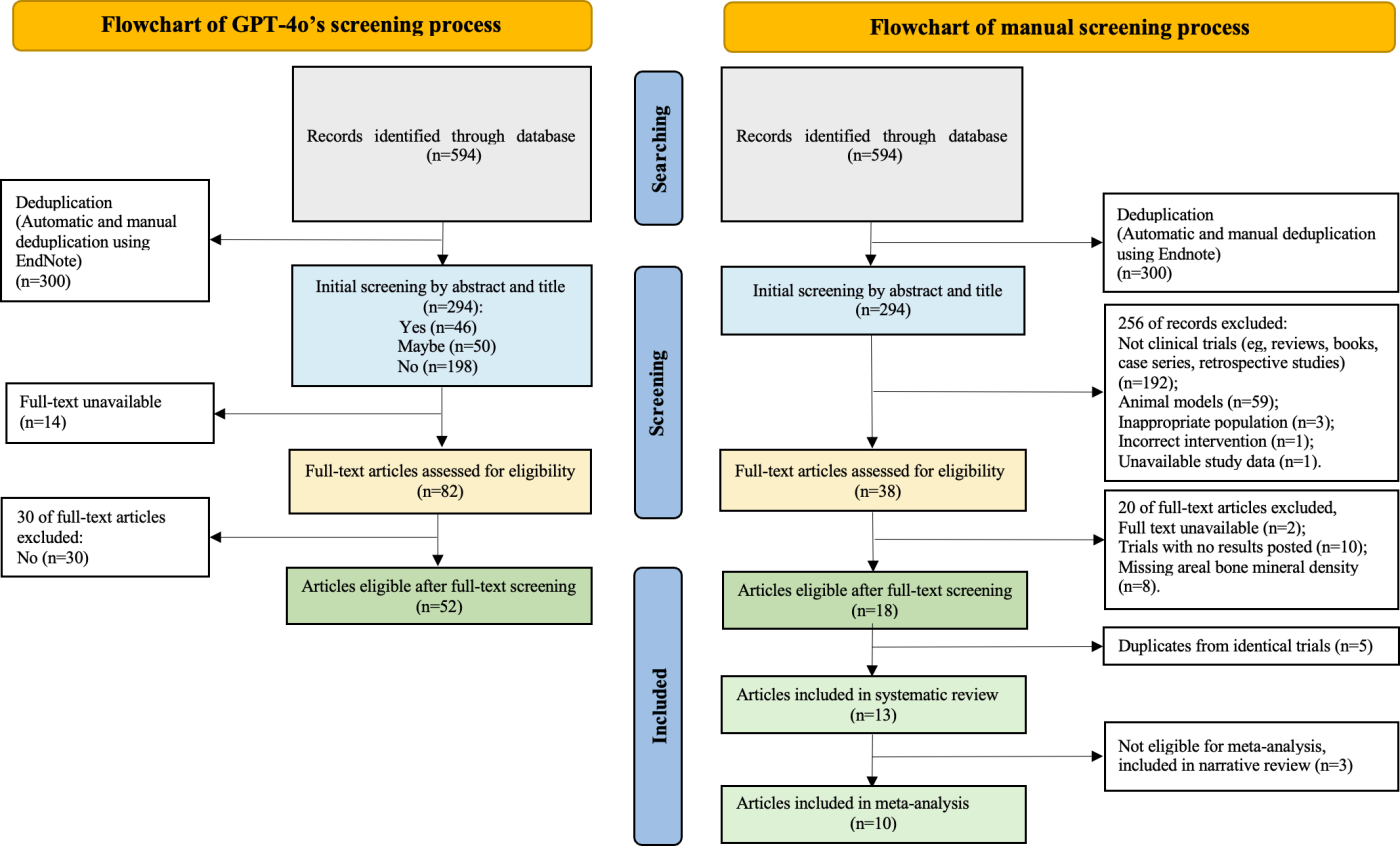
Comparative PRISMA flow diagram for artificial intelligence–assisted versus manual screening in the systematic review of biologics for osteogenesis imperfecta, showing the number of records at each stage and the final set of included trials. PRISMA: Preferred Reporting Items for Systematic Reviews and Meta-Analyses.

The comparison between manual and GPT-based screening is summarized in Figure 1. Both approaches started from the same pool of 594 records, with 294 unique records screened after deduplication. GPT-assisted screening was applied at the report level and classified 52 full-text reports as eligible following title/abstract and full-text assessment, whereas manual screening ultimately included 18 eligible full-text reports from the same pool. Because multiple reports could originate from the same underlying trial, all eligible full-text reports were subsequently mapped to unique studies in a separate, manual study-level consolidation step, in which multiple reports of the same trial were merged. After this consolidation, 13 unique studies were included in the systematic review [14,29-40], of which 10 contributed data to the meta-analysis [29,30,32-34,36-40].

Notably, GPT-based screening dramatically reduced workload, with an average screening time of 3.5 (SD 0.14) seconds per article for title and abstract screening and 9.67 (SD 1.85) seconds for full-text review (Figure S2 in Multimedia Appendix 1), compared to approximately 15 minutes per article for manual screening. This resulted in over 95% total time savings and an approximately 100-fold increase in screening speed per article.

Using manual review as the benchmark, GPT achieved high sensitivity—97.4% for title/abstract screening and 88.9% for full-text review—but lower specificity, at 77% and 87%, respectively, indicating a tendency to favor recall over specificity (Table 1).

**Table 1.** Performance of GPT-4o versus human reviewers in title/abstract and full-text screening: confusion matrices, sensitivity, specificity, and screening time per paper^a^.

	Human					
Document type	Positive	Negative	Total	Sensitivity^b^ (TP/[TP+FN]), %	Specificity^c^ (TN/[TN+FP]), %	Screening time (seconds/paper), mean (SD)
GPT						
Title and abstract				97.4	77	3.50 (0.14)
Positive	37	59	96			
Negative	1	197	198			
Total	38	256	294			
Full text				88.9	87	9.67 (1.85)
Positive	16	36	52			
Negative	2	240	242			
Total	18	276	294			

a The consensus decisions of two independent human reviewers were treated as the gold standard. We explicitly checked whether the large language model (LLM) identified any additional eligible studies that had been missed by both reviewers and found no such cases. This empirical check supports using the human consensus set as the reference standard for evaluating LLM performance in this dataset.

b TP: true positive; FN: false negative.

c TN: true negative; FP: false positive.

### Characteristics of Studies for Systematic Review

A total of 13 trials were included (684 participants) [14,29-40]: 8 controlled studies (4 double-blind trials [29,33,35,39], 2 open-label trials with zoledronate as active comparator [36,37], 1 phase I trial [14], and 1 historical-controlled study [38]), and 5 uncontrolled trials (3 single-arm trials [30,31,34] and 2 open-label, self-controlled trials [32,40]). Interventions included denosumab (n=6), teriparatide (n=3), fresolimumab (n=1), romosozumab (n=1), and setrusumab (n=2), with comparators such as placebo or bisphosphonates. The studies enrolled 355 adults and 329 children and reported outcomes included changes in areal or volumetric BMD, BMD *z* score, fracture incidence, bone turnover markers, and safety profiles. Study characteristics are presented in Table 2, with efficacy outcomes detailed in Table S2 in Multimedia Appendix 1.

**Table 2.** Characteristics of clinical trials of biologics for osteogenesis imperfecta included in the systematic review.

Innovative drug	Author/sponsor (year), location	Study design (follow-up)	OI^a^ type	Diagnosis	Sex (male), n (%), and age (years)	Previous treatment	Group (sample size)^b^	Efficacy	Safety
Denosumab	Liu et al (2024) [37], China	Open-label randomized controlled trial (12 months)	I, III, IV, V	Clinical diagnosis; gene sequencing	54 (64.3%) Age ≤18 years	BPN^c^	N=84 I: DEN^d^ (n=42) C: ZOL^e^ (n=42)	aBMD^f^; BMD^g^ *z* score; BTMs^h^; spine morphometry; fracture incidence	Rebound hypercalcemia; AEs^i^ (overall incidence)
Denosumab	Hoyer-Kuhn et al (2016) [34], Germany	Open-label single-arm pilot trial (12 months)	I-IV	Gene sequencing	7 (70%) Mean age 7 (range 5-11) years	Prior BPN for 2 years and discontinued treatment within 6 months before screening	N=10 I: DEN (n=10) C: N/A^j^	aBMD; BMD *z* score; BTMs; spine morphometry; mobility	Hypocalcemia; arthralgia; aphthous lesion soft palate; muscle pain; pain on the left thoracic side between ribs
Denosumab	Lin et al (2024) [36], China	Open-label randomized controlled trial (12 months)	I, III, IV	Gene sequencing	28 (54.9%) Age ≥18 years	Not received intravenous ZOL within the 12 months before screening	N=51 I: DEN (n=25) C: ZOL (n=26)	aBMD; TBS^k^; BTMs; fracture incidence	Acute-phase reactions; bone pain; overall AEs
Denosumab	Amgen Inc (2022) [30], United States, Australia, Belgium, Bulgaria, Canada, Czechia, France, Germany, Hungary, Italy, Poland, Spain, United Kingdom	Phase 3, prospective, multicenter, single-arm trial (12 months)	I-IV	Clinical diagnosis	80 (52.3%) Mean age 9.3 (SD 3.9; range 2-17) years	N/A	N=153 I: DEN (n=153) C: N/A	BMD *z* score; fracture incidence; growth velocity; questionnaire scores; BTMs	Hypercalcemia; arthralgia; bone pain; discomfort
Denosumab	Rehberg et al (2019) [40], Germany	Phase 2, open-label, self-controlled trial (12 months)	I, III, IV	Gene sequencing	5 (62.5%) Age range 5-10 years	Prior treatment with BPN for at least 2 years, no BPN treatment within 6 months	N=8 I: DEN (n=8) C: N/A	aBMD; TBS	N/A
Denosumab	Mei et al (2025) [38], China^l^	Prospective nonrandomized trial with historical alendronate control (12 months)	I, III, IV, V	Clinical diagnosis; gene sequencing	9 (50%) Pediatrics: mean 9.4 (SD 4.4) years Adults: mean 46.6 (SD 14.6) years	BPN in 8/18 DEN patients (alendronate, ibandronate, and zoledronic acid); historical ALN^m^ controls mainly treatment‑naïve	N=43 I: DEN (n=18) C: ALN (n=25)	BMD; BTMs; fracture incidence; spine morphometry; height velocity	Hypercalcemia; AEs (overall incidence); hyperparathyroidism; hypoparathyroidism; hypocalcemia; hypophosphatemia; arthralgia; muscle pain
Teriparatide	Orwoll et al (2014) [39], United States	Double-blind, placebo-controlled trial (18 months)	I, III, IV	Clinical diagnosis	32 (29.1%) Age ≥18 years	Most never received therapy	N=56 I: TPTD^n^ (n=29) C: Placebo (n=27)	aBMD; BTMs; vBMD^o^; estimated vertebral strength; fracture incidence	No significant differences in adverse events between groups
Teriparatide	Leali et al (2017) [35], Italy, Switzerland	Multicenter, randomized, double-blind, controlled trial (24 months)	I	Clinical diagnosis	N/A Age ≥25 years	No previous skeletal exposure to radiotherapy	N=98 I: TPTD (n=49) C: Neridronate (n=49)	aBMD; BTMs; fracture incidence; pain; quality of life	N/A
Teriparatide	Gatti et al (2013) [32], Italy	Open-label, self-controlled trial (18 months)	I	Clinical diagnosis	N/A Age range 52-72 years	Patients treated with neridronate for at least 2 years discontinued neridronate and were fully reassessed before starting TPTD within 1 month	N=13 I: TPTD (n=13) C: N/A	aBMD; BTMs; Wnt pathway inhibitors	Mild nausea
Fresolimumab	Song et al (2022) [14], United States	Phase I trial (6 months)	III, IV, VIII	Clinical diagnosis	3 (37.5%) Age range 18-55 years	No BPN treatment within 6 months	N=8 I1: FMB^p^ 1 mg/kg (n=4) I2: FMB 4 mg/kg (n=4) C: N/A	aBMD; BTMs	Epistaxis; nausea; malaise; headache; epistaxis; occult blood in urine; bleeding; corrected QT interval prolongation
Romosozumab	El‑Maouche et al (2025) [31], United States, Germany	Phase I trial (3 months)	N/A	Clinical diagnosis	16 (64%) Mean age 10.5 years	N/A	N=25 I: ROM^q^ (n=25) C: N/A	aBMD; BTMs	TEAEs^r^; serious TEAEs; immunogenicity
Setrusumab	Glorieux et al (2024) [33], United States	Phase 2, multicenter, multinational, double-blind, placebo-controlled trial (12 months)	I, III, IV	Clinical diagnosis; gene sequencing	39 (35.5%) Age range 18-74 years	N/A	N=111 I1: SET^s^ 2 mg/kg (n=30) I2: SET 8 mg/kg (n=29) I3: SET 20 mg/kg (n=31) C: Placebo (n=21)	aBMD; vBMD; radial bone strength; BTMs	TEAEs; serious TEAEs; infusion-related reactions
Setrusumab	Nowicki et al (2024) [29], United States	Phase 2, randomized, double-blind, placebo-controlled trial (12 months)	I, III, IV	Clinical diagnosis; gene sequencing	12 (50%) Age range 5-26 years	No treatment with denosumab, teriparatide, romosozumab, growth hormone, or other bone anabolic or antiresorptive medications within 6 months	N=24 I1: 20 mg/kg SET (n=14) I2: 40 mg/kg SET (n=10) C: N/A	aBMD; BMD *z* score; annualized pretreatment fracture rate	TEAEs

a OI: osteogenesis imperfecta.

b N refers to the number of participants who completed the corresponding follow-up period in the original studies.

c BPN: bisphosphonate.

d DEN: denosumab.

e ZOL: zoledronic acid.

f aBMD: areal bone mineral density.

g BMD: bone mineral density.

h BTM: bone turnover marker.

i AE: adverse event.

j Not applicable.

k TBS: trabecular bone score.

l Mean percentage changes in LS aBMD were extracted as reported in the article (33.9% for pediatric and 3.1% for adult subgroups). Standard errors were approximated from group-level baseline and 12-month BMD values assuming a pre-post correlation of 0.7.

m ALN: alendronate.

n TPTD: teriparatide.

o vBMD: volumetric bone mineral density.

p FMB: fresolimumab.

q ROM: romosozumab.

r TEAEs: treatment-emergent adverse events.

s SET: setrusumab.

### Risk of Bias: GPT-4o–Assisted Assessment vs Human Ratings in Studies Included in Meta-Analysis

Manual risk of bias in 10 trials is presented in Figure S3 in Multimedia Appendix 1. Four trials [30,33,34,40] were rated as low risk across all domains. Five trials exhibited a high risk of bias [32,36-39], mainly due to subjective assessment of spinal morphometry in outcome measurement (n=4) [32,36-38], or missing values that could have biased the outcome measurements (n=1) [39]. The overall risk of bias was adjudicated as some concerns for all 10 studies.

Table S1 in Multimedia Appendix 1 compares the risk of bias between human evaluators and the GPT-based tool for the 10 studies. Overall, substantial agreement was observed (weighted κ=0.778, 95% CI 0.710‐0.846) with no significant heterogeneity across studies. All studies except Glorieux et al [33] and Mei et al [38] exceeded the 0.6 threshold for substantial agreement. The greatest discrepancies between human and GPT evaluations were found in the domains of randomization, followed by measurement of the outcome.

### Efficacy of Biologics

#### Age-Adjusted BMD Z-Score Change at Lumbar Spine

##### Denosumab

Three trials [30,34,37] in children reported significant lumbar spine *z* score increases with denosumab at 6 and 12 months. However, the largest existing RCT [37] found no significant advantage of denosumab over zoledronic acid after 12 months (*P*>.05), devaluing the results of the smaller retrospective analysis in Hoyer-Kuhn et al [34] study (n=8), which had reported a greater *z* score gain with denosumab than prior bisphosphonate therapy (mean increase: 1.15 vs 0.31, *P*=.02). A pooled analysis using a meta-analytic approach (though not a formal meta-analysis) of data from Hoyer-Kuhn et al [34] and Amgen Inc [30] yielded a weighted mean *z* score increase of 1 (95% CI 0.811-1.195; n=55) at 12 months.

##### Setrusumab

In the single-arm phase 2 of the ORBIT study (ages 5‐26 years) [29], setrusumab produced significant improvements in lumbar spine BMD *z* scores from baseline (*P*<.001). Pooled across the 20 mg/kg and 40 mg/kg dose groups, mean *z* score increases were 0.85 (SE 0.13) at month 6 and 1.25 (SE 0.17) at month 12, indicating a robust early response. A longer follow-up is needed to determine sustained effects in *z* score.

### Area BMD Percentage Change at Lumbar Spine

**Figure 2.**
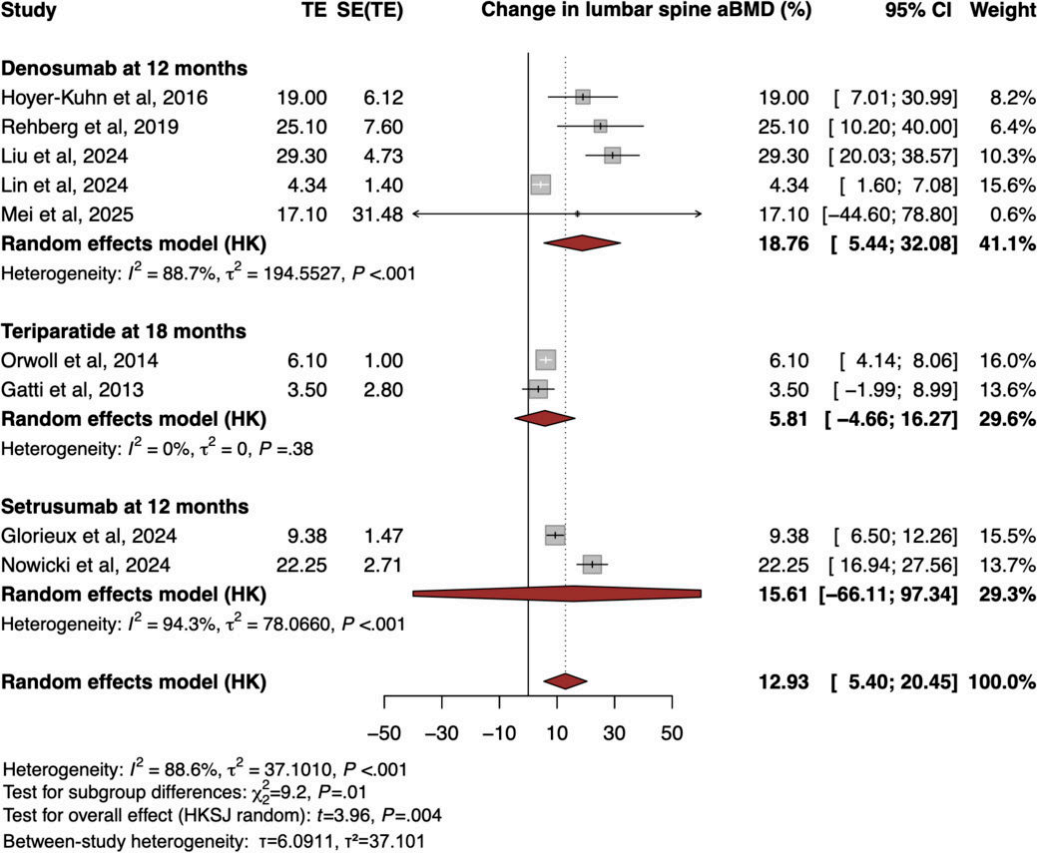
Pooled percentage change in lumbar spine aBMD with different biologic agents in patients with osteogenesis imperfecta. Forest plot from a random-effects meta-analysis (HKSJ method) showing trial-level estimates and pooled effects with 95% CIs for denosumab at 12 months, teriparatide at 18 months, and setrusumab at 12 months. Overall, biologic therapy increased lumbar spine aBMD, although there was substantial between-study heterogeneity. In Mei et al [38], pediatric and adult cohorts were combined within the trial, and only the combined effect was included as a single entry in the primary meta-analysis to avoid double-counting [29,32-34,36-40]. aBMD: areal bone mineral density; HK: Hartung-Knapp method; HKSJ: Hartung-Knapp-Sidik-Jonkman method; SE: standard error; TE: treatment effect.

Twelve trials reported percentage change in lumbar spine aBMD [14,29,31-40], of which 9 trials (180 participants) provided sufficient data for inclusion in the formal meta-analysis (Figure 2) [29,32-34,36-40]. A random-effects model using the HKSJ method was applied, given the clinical and methodological diversity across trials. The pooled percentage increase was 12.93% (95% CI 5.4%‐20.45%). Of the 9 pooled studies, 5 were at high overall risk of bias, mainly due to open-label designs and nonblinded aBMD assessment [32,36-39], potentially introducing subjective measurement influences and additional uncertainty around the pooled effect. Between-study heterogeneity was substantial (*I*²=88.6%, τ²=37.10). Subgroup analyses by drug showed statistically significant differences between treatments (test for subgroup differences: *χ*²_2_=9.2, *P*=.01). Denosumab at 12 months was associated with the largest mean increase (18.76%, 95% CI 5.44%‐32.08%; *I*²=88.7%), whereas teriparatide at 18 months showed a smaller and statistically nonsignificant increase (5.81%, 95% CI −4.66% to 16.27%; *I*²=0%). Setrusumab at 12 months showed a mean increase of 15.61%, but with a very wide and imprecise confidence interval (95% CI −66.11% to 97.34%; *I*²=94.3%), indicating considerable uncertainty about the true treatment effect.

**Figure 3.**
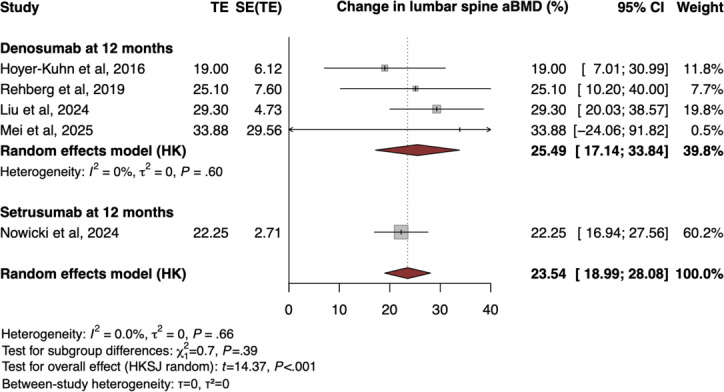
Pediatric subgroup meta-analysis of percentage change from baseline in lumbar spine aBMD in pediatric patients with osteogenesis imperfecta treated with denosumab (12 months) or setrusumab (12 months). The random-effects (HKSJ) forest plot shows trial-level estimates and pooled effects for each agent, indicating that both treatments were associated with increases in lumbar spine aBMD at 12 months with minimal within-agent heterogeneity (denosumab: *I*^2^=0%; setrusumab: single study). There was no evidence of a difference between agents in this pediatric subgroup (test for subgroup differences: *P=.*39) [29,34,37,38,40]*.* aBMD: areal bone mineral density; HK: Hartung-Knapp method; HKSJ: Hartung-Knapp-Sidik-Jonkman method; SE: standard error; TE: treatment effect.

Subgroup analysis showed that in children (Figure 3), the pooled percentage increase in lumbar spine aBMD across the two biologics was 23.54% (95% CI 18.99%‐28.08%; *I*²=0%, τ²=0). Denosumab at 12 months was associated with a mean increase of 25.49% (95% CI 17.14%‐33.84%; *I*²=0%; τ²=0) and setrusumab at 12 months showed a similar effect (22.25%, 95% CI 16.94%‐27.56%; τ²=0). The difference between the two biologics was not statistically significant (test for subgroup differences: *χ*²_2_=0.7, *P*=.39).

**Figure 4.**
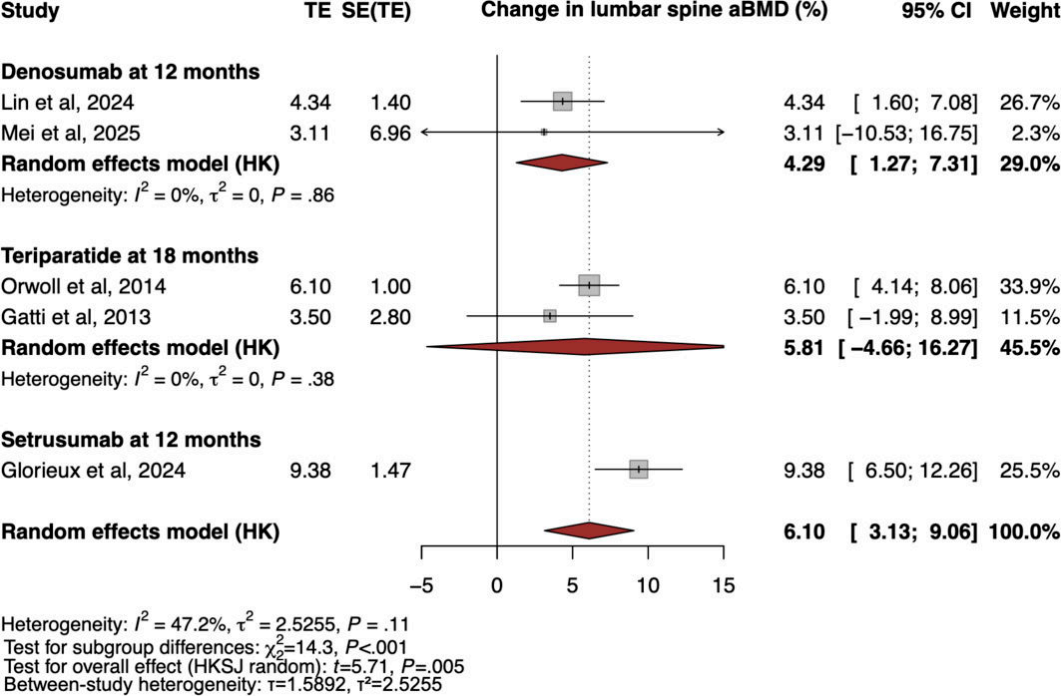
Adult subgroup meta-analysis of percentage change from baseline in lumbar spine aBMD in adult patients with osteogenesis imperfecta treated with denosumab (12 months), teriparatide (18 months), or setrusumab (12 months). The random-effects (HKSJ) forest plot shows trial-level estimates and pooled effects for each agent, indicating that setrusumab is associated with the largest and most precisely estimated increase in aBMD (6.1%, 95% CI 3.13% to 9.06%), whereas denosumab showed a smaller increase (4.29%, 95% CI 1.27% to 7.31%) and teriparatide had a more uncertain pooled estimate (5.81%, 95% CI −4.66% to 16.27%). Evidence for differences across agents was observed (test for subgroup differences *P*<.001) [32,33,36,38,39]. aBMD: areal bone mineral density; HK: Hartung-Knapp method; HKSJ: Hartung-Knapp-Sidik-Jonkman method; SE: standard error; TE: treatment effect.

In adults (Figure 4), the pooled percentage increase in aBMD across the three biologics was 6.1% (95% CI 3.13%‐9.06%; *I*²=47.2%, τ²=2.53), indicating moderate between-study heterogeneity. There was evidence of differences between agents (test for subgroup differences: *χ*²_2_=14.3, *P*<.001). Setrusumab at 12 months produced the largest effect (9.38%, 95% CI 6.5%‐12.26%). In contrast, denosumab at 12 months (4.29%, 95% CI 1.27%‐7.31%; *I*²=0%) and teriparatide at 18 months (5.81%, 95% CI −4.66% to 16.27%) showed smaller and, for teriparatide, statistically uncertain effects. Notably, the setrusumab trial was judged to be at low risk of bias, whereas 4 trials evaluating denosumab and teriparatide were at high risk of bias, adding further uncertainty to these estimates.

### Fracture Reduction

Evidence on fracture reduction with biologics in OI remains limited and mixed. Across 3 trials [30,36,37] and 1 interventional study with historical control [38], denosumab demonstrated fracture rates comparable to bisphosphonates (zoledronic acid and alendronate) and in both adults and children. Intensifying the dosing frequency to every 3 months in children did not yield a lower fracture rate (Q3M: 26.7% vs Q6M: 28.3%; *P*>.05) [30]. For *teriparatide,* 2 adult trials [35,39] found teriparatide (20 μg/day) reduced fracture risk compared to controls, although results were not statistically significant. New fracture rates were lower with teriparatide versus placebo (29% vs 36%, odds ratio 0.73) over 18 months [39] and intravenous neridronate over 24 months (16.3% vs 26.5%; *P*=.10) [35]. A single trial with setrusumab showed dose-dependent fracture reduction [33]: the 20 mg/kg group had the lowest fracture incidence (16.1%) and annualized fracture rate (0.19 per participant-year), compared to 34.5% and 0.54 in the 8 mg/kg group over 12 months.

### Safety of Biologics

Ten studies reported adverse events as safety outcomes [14,29-34,36-38]. Selective outcomes are summarized in Table 3, while all reported outcomes are listed in Table S5 in Multimedia Appendix 1. Metabolic disturbances were notable, with denosumab associated with a high incidence of hypercalcemia (up to 30.95%) and hypercalcemic crisis (14.29%) in children [37]. In adults, denosumab did not increase the risk of hypercalcemia compared to zoledronic acid and significantly reduced the risk of acute-phase reactions (*P*=.002) [36]. Teriparatide showed the highest rate of nausea among adults (53.85%) [32], but serious adverse event rates were similar to placebo or neridronate in head-to-head trials [35,39]. Fresolimumab was associated with medication-related adverse events in all 8 patients with type III OI in a single-arm trial, most commonly epistaxis, though none were classified as serious [14]. Setrusumab emerged as a relatively safe biologic, with no cases of hypercalcemia or treatment-related serious adverse events. Reported adverse events in adults included transient hypocalcemia and a mild headache [33]. Phase 2 trial also confirmed acceptable tolerability in young patients [29]. In a phase 1 pediatric trial of romosozumab, 48% of participants experienced mostly mild-to-moderate treatment-emergent adverse events (TEAEs), with two serious TEAEs (femur and lower-limb fractures) deemed unrelated to treatment [31].

**Table 3.** Top 10 most frequently reported safety outcomes in clinical trials of biologics for osteogenesis imperfecta.

Categories and adverse events	Study (drugs, cases/incidence)
Musculoskeletal disorders	
Arthralgia	Amgen Inc [30] (denosumab): 70/153 (45.7%); Hoyer-Kuhn et al [34] (denosumab): 10 cases; Mei et al [38] (denosumab): 4/8 (50%); Glorieux et al [33] (setrusumab): 1/111 (0.9%); Mei et al [38] (denosumab): 4/8 (50%)
Bone pain	Liu et al [37] (denosumab): 9/42 (21.4%); Amgen Inc [30] (denosumab): 21/153 (13.7%); Lin et al [36] (denosumab): 3/25 (12%); Glorieux et al [33] (setrusumab): 1/111 (0.9%); Nowicki et al [29] (setrusumab): 1/24 (4.2%)
Back pain	Amgen Inc [30] (denosumab): 50/153 (32.7%)
Metabolic disturbances	
Hypocalcemia	Liu et al [37] (denosumab): 1/42 (2.4%); Hoyer-Kuhn et al [34] (denosumab): 1 case; Amgen Inc [30] (denosumab): 15/153 (9.8%); Mei et al [38] (denosumab): 1/8 (12.5%)
Hypercalcemia	Liu et al [37] (denosumab): 13/42 (30.9%); Amgen Inc [30] (denosumab): 28/153 (18.3%); Mei et al [38] (denosumab): 5/8 (62.5%)
Hypercalcemia crisis	Liu et al [37] (denosumab): 6/42 (14.3%)
Gastrointestinal disorders	
Vomiting	Liu et al [37] (denosumab): 6/42 (14.3%); Amgen Inc [30] (denosumab): 13/153 (8.5%)
Nausea	Gatti et al [32] (teriparatide): 7/13 (53.8%)
General disorders	
Infusion/injection site reaction	Glorieux et al [33] (setrusumab): 12/111 (10.81%); Nowicki et al [29] (setrusumab): 7/24 (29.17%); Lin et al [36] (denosumab): 1/25 (4%)
Infections	
Nasopharyngitis	Amgen Inc [30] (denosumab): 23/153 (15.03%)

## Discussion

### Principal Findings

This study addressed the clinical challenges of synthesizing fragmented trial evidence in a rare disease—OI. Clinically, among emerging biologic therapies, denosumab and setrusumab could potentially improve aBMD across age groups, but comparative evidence remains limited and is not yet robust enough to demonstrate clear efficacy superiority or fracture benefits over traditional BPs. To streamline the review process, we also implemented an LLM-assisted, automation-enabled, human-in-the-loop workflow for key steps, including screening and risk of bias assessment. Its outputs showed acceptable agreement with consensus human decisions.

### Novel Biologics for OI: Efficacy and Uncertainty

Patients with OI face substantial unmet needs, with fracture reduction and improved quality of life as central goals [41]. However, most existing studies use changes in aBMD as a surrogate outcome rather than directly assessing clinically meaningful endpoints such as fracture incidence, mobility, and patient-reported measures [42,43]. This is important because a higher aBMD does not always equate to stronger bones in OI [44]. Against this backdrop, our review is the first to systematically evaluate emerging biologics in OI, synthesizing age-specific advantages while underscoring the shared limitations of relying on surrogate endpoints.

In pediatric populations, both denosumab and setrusumab significantly improved lumbar spine aBMD, with denosumab showing the highest overall gain over 12 months. Nevertheless, the largest RCT has not demonstrated a significant advantage of denosumab over intravenous BPs [37], the current standard of care. For setrusumab, early improvements were observed within 12 months, but long-term efficacy and safety remain unconfirmed, as the COSMIC trial (NCT05768854) [45]—the only ongoing phase 3 RCT directly comparing setrusumab and BPs in children over a 24-month period—remains under recruitment. Evidence for fresolimumab in children is lacking, and teriparatide is contraindicated in children due to the acknowledged risk of osteosarcoma [46]. These findings suggest that, while biologics show potential in children, they are not validated for routine use and do not surpass established therapies.

In adults, setrusumab exhibited the greatest increase in lumbar spine aBMD, with higher doses yielding greater gains. This dose-response relationship may be explained by the mechanism proposed by Hosseinitabatabaei et al [47], whereby higher doses stimulate modeling-based bone formation by increasing bone mass directly without requiring prior resorption. This suggests that higher dosing may benefit patients requiring rapid bone accrual. Response to anabolic agents appears subtype-dependent: teriparatide showed efficacy mainly in type I OI [39], with limited benefit in types III and IV, while fresolimumab was effective only in type IV [14], and associated with decreased aBMD in types III and VIII. This means in the adult population, biologics should be considered primarily in the context of clinical trials, with treatment tailored to patient-specific risk profiles and OI subtypes.

Despite aBMD gains with denosumab and setrusumab across age groups, evidence for fracture risk reduction is limited. Denosumab has not shown a significant lower risk in fracture rates versus BPs, even with intensified dosing, which contrasts with findings in osteoporosis [48,49]. This disparity reflects fundamental differences in pathophysiology: in osteoporosis, increased osteoclast activity drives fragility, while in OI, it stems from genetic mutations impairing collagen synthesis. As El-Gazzar et al [50] noted, denosumab reduces bone resorption but does not improve collagen production, folding, or mineralization. Consequently, mineralized material accumulates unevenly within an abnormal collagen matrix, leading to an “over-mineralized but fragile” matrix. Similarly, Rummler et al [51] observed that setrusumab increased bone mass in OI but failed to alter the bone’s multiscale structure, suggesting that fragility arises from other unexplored aspects of bone organization at higher length scales. Also, evidence directly linking specific aBMD changes to fracture risk in OI remains inconclusive. A recent prospective OI cohort did find that lower baseline aBMD (*z* score < −2 SD) predicts a higher fracture risk, but genotype (COL1 splicing/stop/frameshift variants) exerted an even stronger effect, indicating that bone quality and collagen defects may modify the aBMD-fracture relationship [52].

These findings highlight the limitations of aBMD as a surrogate endpoint in OI. Clinically, what matters most to patients is whether biologics reduce fracture rates, improve mobility, and alleviate pain. Future RCTs should therefore prioritize comprehensive bone quality markers—such as the trabecular bone score and microstructural imaging—alongside direct measures of fracture reduction and patient-centered outcomes.

### LLMs in Evidence Synthesis: Reading and Reasoning

#### Literature Screening

This study supports the use of LLMs as effective first-pass screeners to reduce reviewer burden even without domain-specific fine-tuning. During screening, GPT-4o was calibrated to prioritize sensitivity, suggesting that LLMs can substantially ease the initial workload by flagging potentially eligible studies despite complex and heterogeneous clinical trial designs—an approach that is often desirable in rare-disease reviews, where missing a key trial is costly.

However, specificity was relatively low during title and abstract screening, reflecting a conservative approach to limited or incomplete information. GPT-4o was instructed to include studies marked as “yes” or “maybe,” with “maybe” indicating insufficient information for confident exclusion. This over-inclusive approach at the initial stage, with strict exclusion deferred to full-text review, mirrors established logic in human screening workflows [53].

This “high-recall, low-specificity” pattern is consistent with the metrics reported by Dennstädt et al [54], where several openly available LLMs achieved ≥95%‐100% sensitivity but often <30% specificity when lenient thresholds were chosen to minimize false negatives. Our screening setup operated in the same sensitivity-oriented regime: GPT-4o over-included 96 studies in the first round, and it successfully identified all 13 human final-selected studies. Thus, the lower specificity is better viewed as a precautionary trade-off that prioritizes recall in the face of ambiguity.

In the full-text phase, GPT-4o produced only one false negative by erroneously excluding an eligible open-label, single-arm trial. Despite inclusion criteria explicitly permitting such designs, the model inferred—based on surrounding context—that open-label studies should involve two arms like RCTs. This reflects a pattern-based generalization bias [55], whereby the model applies common associations from its training data to oversimplify text instead of strictly adhering to the stated criteria. This highlights the importance of precise, detailed instructions tailored to specific review needs—such as the types of trial included—to help the model interpret nuanced criteria accurately.

#### Risk of Bias Assessment

GPT-4o achieved substantial agreement with human raters in risk assessment, but performance varied across domains, reflecting differences in the cognitive demands of each task. In the randomization domain, the model frequently inferred “yes” based on indirect contextual hints—such as a study being labeled “prospective RCT” or conducted at a well-known hospital—even when allocation concealment was not described. This reflects an optimism bias [56], whereby the model systematically overestimates the value despite lacking concrete evidence. In the measurement domain, a positional (salience) bias was observed [57]: the model assigned a low risk of bias whenever any objective outcome (eg, laboratory tests) was reported, even if subjective outcomes or lack of assessor blinding were also present. This refers to a tendency to overweight prominent or easily identifiable features while overlooking less visible but equally important factors.

Although these biases differ across tasks—overgeneralizing exclusion during full-text screening and over-crediting judging during risk assessment—they share a common root: LLMs rely on probabilistic heuristics derived from linguistic patterns rather than structured, context-sensitive reasoning [58]. As a result, current models excel at information extraction but struggle with tasks that require skepticism and uncertainty handling. This limitation becomes particularly evident in scenarios where missing information must be interpreted cautiously [59].

Looking ahead, we expect future models to move beyond better reading toward better reasoning—questioning unclear points before operation, closely adhering to review standards, and aligning more tightly with domain specifics across clinical subfields to reduce overgeneralization. From a user perspective, prompt engineering (eg, structured templates and explicit decision rules) can help mitigate bias. In practice, we recommend a hybrid approach: LLMs should assist, but not replace, human judgment, especially in tasks like full-text screening and quality assessment, where structured skepticism and contextual discernment remain essential.

### Limitations

This study has several limitations. First, the limited number of eligible trials, particularly for romosozumab and fresolimumab, precluded pooled analyses and publication bias assessments. Second, the short duration of included studies (≤24 months) limits insights into long-term efficacy, safety, and rebound effects, which is crucial given OI typically necessitates lifelong pharmacological treatment. Third, clinical heterogeneity from variations in OI subtypes, doses, and prior BP use, together with substantial design heterogeneity and limited comparative data, complicates quantitative cross-trial comparisons and precludes a formal certainty-of-evidence assessment (eg, GRADE). Fourth, all prompts were administered to models without prior domain-specific fine-tuning; therefore, our findings may not reflect the full potential of LLMs further trained or adapted specifically for systematic review tasks. Finally, our search was restricted to English-language publications, which may have introduced language bias and led to the omission of relevant non-English studies. In addition, we relied solely on free-text terms in titles, abstracts, and keywords, and did not use controlled vocabulary (eg, MeSH/Emtree), which may have reduced the sensitivity of the search and resulted in missed studies. However, reference lists of included trials were also hand-searched for additional eligible studies, which may have partially mitigated the risk of missing relevant evidence. Given that this review provides some of the first evidence on the use of AI in a rare genetic disorder with a relatively small evidence base, future research should replicate similar AI-assisted workflows in scientific fields with larger and more complex datasets to determine how well AI performs in reviews involving larger evidence bases.

### Implications and Conclusions

This review is distinctive in that it both synthesizes the fragmented trial evidence on emerging biologics in OI and prospectively evaluates an LLM-assisted workflow that combines screening and risk-of-bias assessment against consensus human judgments. Unlike prior reviews that focus either on pharmacologic efficacy or AI methods, we provide a unified, quantitative evaluation of both clinical effects and AI performance within a real-world rare-disease review. Taken together, our findings suggest that biologics should remain largely confined to clinical trials until fracture and safety outcomes are better established, while LLM-based tools can be used to reduce reviewer workload under structured human oversight and to inform the integration of LLM-assisted evidence surveillance into “living” rare-disease guidelines (eg, automated literature monitoring followed by targeted human appraisal). At a policy level, these findings highlight the need for governance frameworks that define minimum performance standards, transparency requirements, and human-oversight safeguards for AI-driven updates to rare-disease clinical guidance.

In conclusion, denosumab and setrusumab show potential efficacy in improving aBMD across age groups in patients with common COL1-related OI types (I-IV), yet evidence for fracture reduction remains inconclusive. Integrating biologics into clinical practice will require more rigorous, long-term RCTs, especially in children. LLM-based tools can improve evidence synthesis in rare diseases by integrating fragmented and heterogeneous clinical trial data. Refined prompts that support context-aware reasoning are essential for trustworthy AI, enabling LLMs to automate rule-based tasks under instruction-based workflows.

## Supplementary material

10.2196/85840 Multimedia Appendix 1 Additional methods, risk-of-bias criteria, and detailed trial results.

10.2196/85840 Multimedia Appendix 2 Prompts screening.

10.2196/85840 Multimedia Appendix 3 Risk of bias assessments.

10.2196/85840 Checklist 1 PRISMA 2020 expanded checklist.

10.2196/85840 Checklist 2 PRISMA-S Checklist.

## Abbreviations

aBMD: areal bone mineral density
AI: artificial intelligence
BMD: bone mineral density
BPs: bisphosphonates
HKSJ: Hartung-Knapp-Sidik-Jonkman method
LLM: large language model
OI: osteogenesis imperfecta
PRISMA: Preferred Reporting Items for Systematic Reviews and Meta-Analyses
RCT: randomized controlled trial
RoB 2: revised Cochrane risk-of-bias tool for randomized trials
TEAE: treatment-emergent adverse event

## References

[1] Sillence DO, Senn A, Danks DM (1979). Genetic heterogeneity in osteogenesis imperfecta. J Med Genet.

[2] Bishop N (2010). Characterising and treating osteogenesis imperfecta. Early Hum Dev.

[3] Cundy T (2012). Recent advances in osteogenesis imperfecta. Calcif Tissue Int.

[4] Forlino A, Cabral WA, Barnes AM, Marini JC (2011). New perspectives on osteogenesis imperfecta. Nat Rev Endocrinol.

[5] Sun Y, Li L, Wang J, Liu H, Wang H (2024). Emerging landscape of osteogenesis imperfecta pathogenesis and therapeutic approaches. ACS Pharmacol Transl Sci.

[6] Marini JC, Forlino A, Bächinger HP (2017). Osteogenesis imperfecta. Nat Rev Dis Primers.

[7] Tosteson ANA, Grove MR, Hammond CS (2003). Early discontinuation of treatment for osteoporosis. Am J Med.

[8] Antoniazzi F, Zamboni G, Lauriola S, Donadi L, Adami S, Tatò L (2006). Early bisphosphonate treatment in infants with severe osteogenesis imperfecta. J Pediatr.

[9] Shi CG, Zhang Y, Yuan W (2016). Efficacy of bisphosphonates on bone mineral density and fracture rate in patients with osteogenesis imperfecta: a systematic review and meta-analysis. Am J Ther.

[10] Datir RR, Datir RR, Datir PR, Heyrani N (2025). A systematic review on the efficacy of bisphosphonates on osteogenesis imperfecta. Cureus.

[11] Majdoub F, Ferjani HL, Nessib DB, Kaffel D, Maatallah K, Hamdi W (2023). Denosumab use in osteogenesis imperfecta: an update on therapeutic approaches. Ann Pediatr Endocrinol Metab.

[12] Marini F, Giusti F, Palmini G, Brandi ML (2023). Role of Wnt signaling and sclerostin in bone and as therapeutic targets in skeletal disorders. Osteoporos Int.

[13] Liu W, Nicol L, Orwoll E (2024). Current and developing pharmacologic agents for improving skeletal health in adults with osteogenesis imperfecta. Calcif Tissue Int.

[14] Song IW, Nagamani SC, Nguyen D (2022). Targeting TGF-β for treatment of osteogenesis imperfecta. J Clin Invest.

[15] Cierco Jimenez R, Lee T, Rosillo N (2022). Machine learning computational tools to assist the performance of systematic reviews: a mapping review. BMC Med Res Methodol.

[16] Wang Z, Nayfeh T, Tetzlaff J, O’Blenis P, Murad MH (2020). Error rates of human reviewers during abstract screening in systematic reviews. PLoS One.

[17] Wang S, Scells H, Koopman B, Zuccon G (2023). Can ChatGPT write a good Boolean query for systematic review literature search?. arXiv.

[18] Oami T, Okada Y, Nakada TA (2024). Performance of a large language model in screening citations. JAMA Netw Open.

[19] Khraisha Q, Put S, Kappenberg J, Warraitch A, Hadfield K (2024). Can large language models replace humans in systematic reviews? Evaluating GPT-4’s efficacy in screening and extracting data from peer-reviewed and grey literature in multiple languages. Res Synth Methods.

[20] Scherbakov D, Hubig N, Jansari V, Bakumenko A, Lenert LA (2025). The emergence of large language models as tools in literature reviews: a large language model-assisted systematic review. J Am Med Inform Assoc.

[21] Xu S, Zhao Z, Liu X, Meng XL (2025). A comparative study of screening performance between Abstrackr and GPT models: systematic review and contextual analysis. BMC Med Inform Decis Mak.

[22] Alshami A, Elsayed M, Ali E, Eltoukhy AEE, Zayed T (2023). Harnessing the power of ChatGPT for automating systematic review process: methodology, case study, limitations, and future directions. Systems.

[23] Cai X, Geng Y, Du Y (2025). Utilizing large language models to select literature for meta-analysis shows workload reduction while maintaining a similar recall level as manual curation. BMC Med Res Methodol.

[24] Page MJ, McKenzie JE, Bossuyt PM (2021). The PRISMA 2020 statement: an updated guideline for reporting systematic reviews. BMJ.

[25] Rethlefsen ML, Kirtley S, Waffenschmidt S (2021). PRISMA-S: an extension to the PRISMA statement for reporting literature searches in systematic reviews. Syst Rev.

[26] Sterne JAC, Savović J, Page MJ (2019). RoB 2: a revised tool for assessing risk of bias in randomised trials. BMJ.

[27] McHugh ML (2012). Interrater reliability: the kappa statistic. Biochem Med.

[28] IntHout J, Ioannidis JPA, Borm GF (2014). The Hartung-Knapp-Sidik-Jonkman method for random effects meta-analysis is straightforward and considerably outperforms the standard DerSimonian-Laird method. BMC Med Res Methodol.

[29] (2025). World Congress on Osteoporosis, Osteoarthritis and Musculoskeletal Diseases (WCO-IOF-ESCEO 2025). Aging Clin Exp Res.

[30] (2015). Multicenter, single-arm study to evaluate efficacy, safety, & pharmacokinetics of denosumab in children w/ OI (OI). ClinicalTrials.gov.

[31] El-Maouche D, Jeswani R, Sohn W, Zhou L, Semler O (2025). SUN-718 results from a phase 1, open-label, ascending multiple-dose study to evaluate safety, tolerability, pharmacokinetics, and pharmacodynamics of romosozumab in children and adolescents with osteogenesis imperfecta and phase 3 study rationale. Journal of the Endocrine Society.

[32] Gatti D, Rossini M, Viapiana O (2013). Teriparatide treatment in adult patients with osteogenesis imperfecta type I. Calcif Tissue Int.

[33] Glorieux FH, Langdahl B, Chapurlat R (2024). Setrusumab for the treatment of osteogenesis imperfecta: 12-month results from the phase 2b asteroid study. J Bone Miner Res.

[34] Hoyer-Kuhn H, Franklin J, Allo G (2016). Safety and efficacy of denosumab in children with osteogenesis imperfecta—first prospective trial. J Musculoskelet Neuronal Interact.

[35] Leali PT, Balsano M, Maestretti G (2017). Efficacy of teriparatide *vs* neridronate in adults with osteogenesis imperfecta type I: a prospective randomized international clinical study. Clin Cases Miner Bone Metab.

[36] Lin X, Hu J, Zhou B (2024). Efficacy and safety of denosumab vs zoledronic acid in OI adults: a prospective, open-label, randomized study. J Clin Endocrinol Metab.

[37] Liu J, Lin X, Sun L (2024). Safety and efficacy of denosumab in children with osteogenesis imperfecta—the first prospective comparative study. J Clin Endocrinol Metab.

[38] Mei Y, Cai S, Jiang Y (2025). Denosumab in patients with osteogenesis imperfecta and a historical control study with alendronate. Front Endocrinol (Lausanne).

[39] Orwoll ES, Shapiro J, Veith S (2014). Evaluation of teriparatide treatment in adults with osteogenesis imperfecta. J Clin Invest.

[40] Rehberg M, Winzenrieth R, Hoyer-Kuhn H, Duran I, Schoenau E, Semler O (2019). TBS as a tool to differentiate the impact of antiresorptives on cortical and trabecular bone in children with osteogenesis imperfecta. J Clin Densitom.

[41] Aubry-Rozier B, Richard C, Unger S (2020). Osteogenesis imperfecta: towards an individualised interdisciplinary care strategy to improve physical activity and quality of life. Swiss Med Wkly.

[42] Hosseinitabatabaei S, McCluskey S, Denton C (2025). Bone microstructural and strength changes over one year in children with osteogenesis imperfecta are comparable to age- and sex-matched healthy controls. J Bone Miner Res.

[43] Patel RM, Nagamani SCS, Cuthbertson D (2015). A cross-sectional multicenter study of osteogenesis imperfecta in North America – results from the linked clinical research centers. Clin Genet.

[44] Rauch F, Land C, Cornibert S, Schoenau E, Glorieux FH (2005). High and low density in the same bone: a study on children and adolescents with mild osteogenesis imperfecta. Bone.

[45] (2023). An open-label, randomized, active-controlled, phase 3 study of setrusumab compared with bisphosphonates in pediatric subjects with osteogenesis imperfecta types I, III or IV. https://clinicaltrials.gov/study/NCT05768854.

[46] Neer RM, Arnaud CD, Zanchetta JR (2001). Effect of parathyroid hormone (1-34) on fractures and bone mineral density in postmenopausal women with osteoporosis. N Engl J Med.

[47] Hosseinitabatabaei S, Vitienes I, Rummler M, Birkhold A, Rauch F, Willie BM (2025). Non-invasive quantification of bone (re)modeling dynamics in adults with osteogenesis imperfecta treated with setrusumab using timelapse high-resolution peripheral-quantitative computed tomography. J Bone Miner Res.

[48] Adler RA, Gill RS (2011). Clinical utility of denosumab for treatment of bone loss in men and women. Clin Interv Aging.

[49] Cummings SR, San Martin J, McClung MR (2009). Denosumab for prevention of fractures in postmenopausal women with osteoporosis. N Engl J Med.

[50] El-Gazzar AAO, Högler WAO (2021). Mechanisms of bone fragility: from osteogenesis imperfecta to secondary osteoporosis. Int J Mol Sci.

[51] Rummler M, Schemenz V, McCluskey S (2024). Bone matrix properties in adults with osteogenesis imperfecta are not adversely affected by setrusumab—a sclerostin neutralizing antibody. J Bone Miner Res.

[52] Blandin C, Collet C, Ostertag A, Funck-Brentano T, Cohen-Solal M (2025). Genetics and bone mineral density predict the fractures in adults with osteogenesis imperfecta: a prospective study. J Clin Endocrinol Metab.

[53] Lefebvre C, Briscoe S, Featherstone R, Littlewood A, Metzendorf MI, Higgins JP, Thomas J, Chandler J, Cumpston M, Li T (2025). Cochrane Handbook for Systematic Reviews of Interventions Version 651.

[54] Dennstädt F, Zink J, Putora PM, Hastings J, Cihoric N (2024). Title and abstract screening for literature reviews using large language models: an exploratory study in the biomedical domain. Syst Rev.

[55] Peters U, Chin-Yee B (2025). Generalization bias in large language model summarization of scientific research. R Soc Open Sci.

[56] Li X, Feng H, Yang H, Huang J (2024). Can ChatGPT reduce human financial analysts’ optimistic biases?. Econ Polit Stud.

[57] Yu T (2025). Benchmarking reasoning robustness in large language models. arXiv.

[58] Bender EM (2021). Proceedings of the 2021 ACM Conference on Fairness, Accountability, and Transparency.

[59] Huang Y, Song J, Wang Z (2023). Look before you leap: an exploratory study of uncertainty measurement for large language models. arXiv.

[60] Suchikova Y, Tsybuliak N, Teixeira da Silva JA, Nazarovets S (2026). GAIDeT (Generative AI Delegation Taxonomy): a taxonomy for humans to delegate tasks to generative artificial intelligence in scientific research and publishing. Account Res.

