# Supplementary material for: Artificial Intelligence for Evidence Synthesis of Emerging Biologics to Improve Skeletal Health in Osteogenesis Imperfecta: Systematic Review and Meta-Analysis
Source: J Med Internet Res. 2026 Jul 10;28:e85840. doi: 10.2196/85840 (PMC13354119; doi:10.2196/85840)
Supplement: Multimedia Appendix 1 [file jmir-v28-e85840-s001.pdf]

Multimedia Appendix 1. Additional Methods, Risk-of-Bias Criteria, and Detailed Trial Results

Contents

Supplementary Methods
Table S1. Database search strategies for innovative biologic interventions in osteogenesis imperfecta ………….……………………...………..p. S2
Table S2. Inclusion and exclusion criteria for title/abstract and full-text screening…………...…………………………..………………….....p. S3
S3. Cochrane Risk of Bias 2 (RoB 2) signalling questions and adapted criteria for single-arm trials ………………………………………….p. S4

Supplementary Figures
Figure S1(a-e). Subgroup meta-analyses of percentage change of areal bone mineral density (aBMD) by original study design ……..…….p. S10
Figure S2. Comparison of GPT-4o screening time for full-text versus title/abstract documents ……………………………………………...p. S15
Figure S3. Risk-of-bias assessment of trials included in the meta-analysis using an adapted RoB 2 tool ……………………………………p. S16

Supplementary Tables
Table S3. Quality assessment results of human and GPT-based tools ……………………………………..……………………………..p. S17
Table S4. Efficacy outcomes of included trials of innovative biologic therapies in osteogenesis imperfecta ………………………………... p. S18
S4a. Supplementary qualitative synthesis of efficacy outcomes…………………………………….………………………………….…p. S30
Table S5. Safety outcomes in trials reporting adverse events ………………………………………………………………………………p. S31

References……………………………………..……………………………………………………….…………………………………p. S35

➢ Supplementary Methods

Table S1 Database search strategies for innovative biologic interventions in osteogenesis imperfecta

Database	Coverage period	Search strategy (Boolean query)	Records identified	Records after Endnote deduplication
PubMed	From inception to 2025-12-01	(((((osteogenesis imperfecta[Title/Abstract]) OR (fragilitas ossium[Title/Abstract])) OR (dysostosis[Title/Abstract])) OR (osteopsathyrosis[Title/Abstract])) OR (brittle bone disease[Title/Abstract]) AND (1950:2025/12/2[pdat]))<br>**AND**<br>(((((((((denosumab) OR (teriparatide)) OR (fresolimumab)) OR (romosozumab)) OR (setrusumab)) OR (blosozumab)) OR (BPS804)) OR (TST002)) OR (somatropin) AND (1950:2025/12/2[pdat])) AND (1000/1/1:2025/12/2[pdat])	127	101
ScienceDirect	From inception to 2025-12-01	Title, abstract or author-specified keywords: osteogenesis imperfecta OR fragilitas ossium OR dysostosis OR osteopsathyrosis OR brittle bone disease<br>**AND**<br>Find articles with these terms: teriparatide OR fresolimumab OR setrusumab OR BPS804 OR romosozumab OR blosozumab OR TST002 OR somatropin OR denosumab	44	24
Embase	From inception to 2025-12-01	(osteogenesis imperfecta or fragilitas ossium or dysostosis or osteopsathyrosis or brittle bone disease).ab.<br>**AND**<br>(teriparatide or fresolimumab or setrusumab or BPS804 or romosozumab or blosozumab or TST002 or somatropin or denosumab).af.	244	184
Web of science	From inception to 2025-12-01	((((AB=(osteogenesis imperfecta)) OR AB=(fragilitas ossium)) OR AB=(dysostosis)) OR AB=(osteopsathyrosis)) OR AB=(brittle bone disease)<br>**AND**<br>((((((((ALL=(Denosumab)) OR ALL=(teriparatide)) OR ALL=(fresolimumab)) OR ALL=(romosozumab)) OR ALL=(setrusumab)) OR ALL=(blosozumab)) OR ALL=(somatropin)) OR ALL=(BPS804)) OR ALL=(TST002)	142	75
Cochrane Library (CENTRAL)	From inception to 2025-12-01	(osteogenesis imperfecta OR fragilitas ossium OR dysostosis OR osteopsathyrosis OR brittle bone disease):ti,ab,kw<br>**AND**<br>(teriparatide OR fresolimumab OR setrusumab OR BPS804 OR romosozumab OR blosozumab OR TST002 OR somatropin OR denosumab):ti,ab,kw" (Word variations have been searched)	37	27

Note: The full Boolean search strings for each database are reported exactly as implemented, including field tags and truncation symbols.

Table S2 Inclusion and exclusion criteria at the title/abstract and full-text screening stages

Screening Stage	Domain	Inclusion criteria	Exclusion criteria
Titles/Abstracts			
	Study design	Eligible study designs included randomized controlled trials (RCTs), non-randomized controlled trials (non-RCTs), quasi-randomized trials, crossover trials, prospective interventional open-label trials (including single-arm studies), and historical-control interventional trials.	Case reports, books, or reviews.
	Populations	All types and ages of patients with OI were diagnosed by genetic diagnosis or by clinical criteria consistent with recognized guidelines.	Animal studies
	Interventions	Innovative medicines, specifically including teriparatide, fresolimumab (TGF-β), setrusumab (BPS804), romosozumab, blosozumab (TST002), somatropin, and denosumab.	
	Outcomes	Report at least one the efficacy outcomes of the biologics	
	Control	Comparators included bisphosphonates, placebo, different doses of the same investigational drug, or other approved or investigational biological agents not listed above; or no treatment;	
Full texts			
	Populations	Same as title/abstract screening.	
	Interventions	Same as title/abstract screening.	
	Outcomes	Report at least one quantified efficacy outcome, particularly changes in areal bone mineral density (aBMD) and/or fracture incidence.	No quantitative efficacy data (eg, narrative reports only, no extractable aBMD or fracture outcomes).
	Control	Same as title/abstract screening.	

Note: Studies for which eligibility could not be determined at the title/abstract screening stage were automatically carried forward to full-text screening.

Signalling Questions from Cochrane Rob 2 Tool and Adapted Criteria for Single-Arm Trials (Marked in Yellow)

Domain 1: Risk of bias arising from the randomization process

**Signalling questions**	**Adaptions for Single arm trial**	**Response options**
**1.1 Was the allocation sequence random?**	**1.1 Were participants enrolled consecutively or used objective criteria to minimize selection bias?**	Y / PY / PN / N / NI
**1.2 Was the allocation sequence concealed until participants were enrolled and assigned to interventions?**	**1.2 Were participants enrolled without knowledge of study intervention (single arm trial) or other biases that could influence inclusion?**	Y / PY / PN / N / NI
**1.3 Did baseline differences between intervention groups suggest a problem with the randomization process?**	**1.3 Were baseline characteristics of participants clearly reported, and were participants representative of the target population?**	Y / PY / PN / N / NI
**Risk-of-bias judgement**		Low / High / Some concerns
Optional: What is the predicted direction of bias arising from the randomization process?		NA / Favours experimental / Favours comparator / Towards null /Away from null / Unpredictable

Domain 2: Risk of bias due to deviations from the intended interventions (effect of assignment to intervention)

Signalling questions	Adaptions for Single arm trial	Response options
**2.1 Were participants aware of their assigned intervention during the trial?**		Y / PY / PN / N / NI
**2.2 Were carers and people delivering the interventions aware of participants' assigned intervention during the trial?**		Y / PY / PN / N / NI
**2.3 If Y/PY/NI to 2.1 or 2.2: Were there deviations from the intended intervention that arose because of the trial context?**		NA / Y / PY / PN / N / NI
**2.4 If Y/PY to 2.3: Were these deviations likely to have affected the outcome?**		NA / Y / PY / PN / N / NI
**2.5 If Y/PY/NI to 2.4: Were these deviations from intended intervention balanced between groups?**	**2.5 If Y/PY/NI to 2.4: To what extent could these deviations from the intended intervention affect the interpretation of the study outcomes (e.g. through systematic or clinically relevant differences in exposure)?**	NA / Y / PY / PN / N / NI
**2.6 Was an appropriate analysis used to estimate the effect of assignment to intervention?**	**2.6 Were appropriate statistical analyses used to evaluate changes in outcomes over time or determine the effect of the intervention?**	Y / PY / PN / N / NI
**2.7 If N/PN/NI to 2.6: Was there potential for a substantial impact (on the result) of the failure to analyse participants in the group to which they were randomized?**	**2.7 If N/PN/NI to 2.6: Was there potential for a substantial impact on the results due to inadequate evaluation of outcome changes or incorrect estimation of the intervention effect?**	NA / Y / PY / PN / N / NI
**Risk-of-bias judgement**		Low / High / Some concerns
Optional: What is the predicted direction of bias due to deviations from intended interventions?		NA / Favours experimental / Favours comparator / Towards null /Away from null / Unpredictable

Domain 3: Missing outcome data

Signalling questions	Adaptions for Single arm trial	Response options
**3.1 Were data for this outcome available for all, or nearly all, participants randomized?**		Y / PY / PN / N / NI
**3.2 If N/PN/NI to 3.1: Is there evidence that the result was not biased by missing outcome data?**		NA / Y / PY / PN / N
**3.3 If N/PN to 3.2: Could missingness in the outcome depend on its true value?**		NA / Y / PY / PN / N / NI
**3.4 If Y/PY/NI to 3.3: Is it likely that missingness in the outcome depended on its true value?**		NA / Y / PY / PN / N / NI
**Risk-of-bias judgement**		Low / High / Some concerns
Optional: What is the predicted direction of bias due to missing outcome data?		NA / Favours experimental / Favours comparator / Towards null /Away from null / Unpredictable

Domain 4: Risk of bias in measurement of the outcome

Signalling questions	Adaptions for Single arm trial	Response options
**4.1 Was the method of measuring the outcome inappropriate?**		Y / PY / PN / N / NI
**4.2 Could measurement or ascertainment of the outcome have differed between intervention groups?**		Y / PY / PN / N / NI
**4.3 If N/PN/NI to 4.1 and 4.2: Were outcome assessors aware of the intervention received by study participants?**	**4.3 Were outcome assessors aware of the intervention received by study participants?**	NA / Y / PY / PN / N / NI
**4.4 If Y/PY/NI to 4.3: Could assessment of the outcome have been influenced by knowledge of intervention received?**	**4.4 Could assessment of the outcome have been influenced by knowledge of the intervention received (due to the open-label design)?**	NA / Y / PY / PN / N / NI
**4.5 If Y/PY/NI to 4.4: Is it likely that assessment of the outcome was influenced by knowledge of intervention received?**	**4.5 Is it likely that assessment of the outcome was influenced by knowledge of the intervention received (due to the open-label design)?**	NA / Y / PY / PN / N / NI
**Risk-of-bias judgement**		Low / High / Some concerns
Optional: What is the predicted direction of bias in measurement of the outcome?		NA / Favours experimental / Favours comparator / Towards null /Away from null / Unpredictable

Domain 5: Risk of bias in selection of the reported result

Signalling questions	Adaptions for Single arm trial	Response options
**5.1 Were the data that produced this result analysed in accordance with a pre-specified analysis plan that was finalized before unblinded outcome data were available for analysis?**		Y / PY / PN / N / NI
**Is the numerical result being assessed likely to have been selected, on the basis of the results, from...**		
**5.2. ... multiple eligible outcome measurements (e.g. scales, definitions, time points) within the outcome domain?**		Y / PY / PN / N / NI
**5.3 ... multiple eligible analyses of the data?**		Y / PY / PN / N / NI
**Risk-of-bias judgement**		Low / High / Some concerns
Optional: What is the predicted direction of bias due to selection of the reported result?		NA / Favours experimental / Favours comparator / Towards null /Away from null / Unpredictable

Overall risk of bias

**Risk-of-bias judgement**		Low / High / Some concerns
Optional: What is the overall predicted direction of bias for this outcome?		NA / Favours experimental / Favours comparator / Towards null /Away from null / Unpredictable

Abbreviations: Y, Yes; PY, Probably Yes; PN, Probably No; N, No; NI, No Information.

➢ Supplementary Figures

Figure S1a Pooled percentage change from baseline in lumbar spine areal bone mineral density (aBMD) following biologic therapy in patients with osteogenesis imperfecta, restricted to non-randomized evidence (single-arm and/or self-controlled studies; no RCTs included) [29,32-34,38,40]. Forest plot from a random-effects meta-analysis using the Hartung–Knapp–Sidik–Jonkman method, showing study-level estimates and pooled effects with 95% confidence intervals for denosumab at 12 months, teriparatide at 18 months, and setrusumab at 12 months. Overall, biologic therapy was associated with increased lumbar spine aBMD, although substantial between-study heterogeneity was observed.

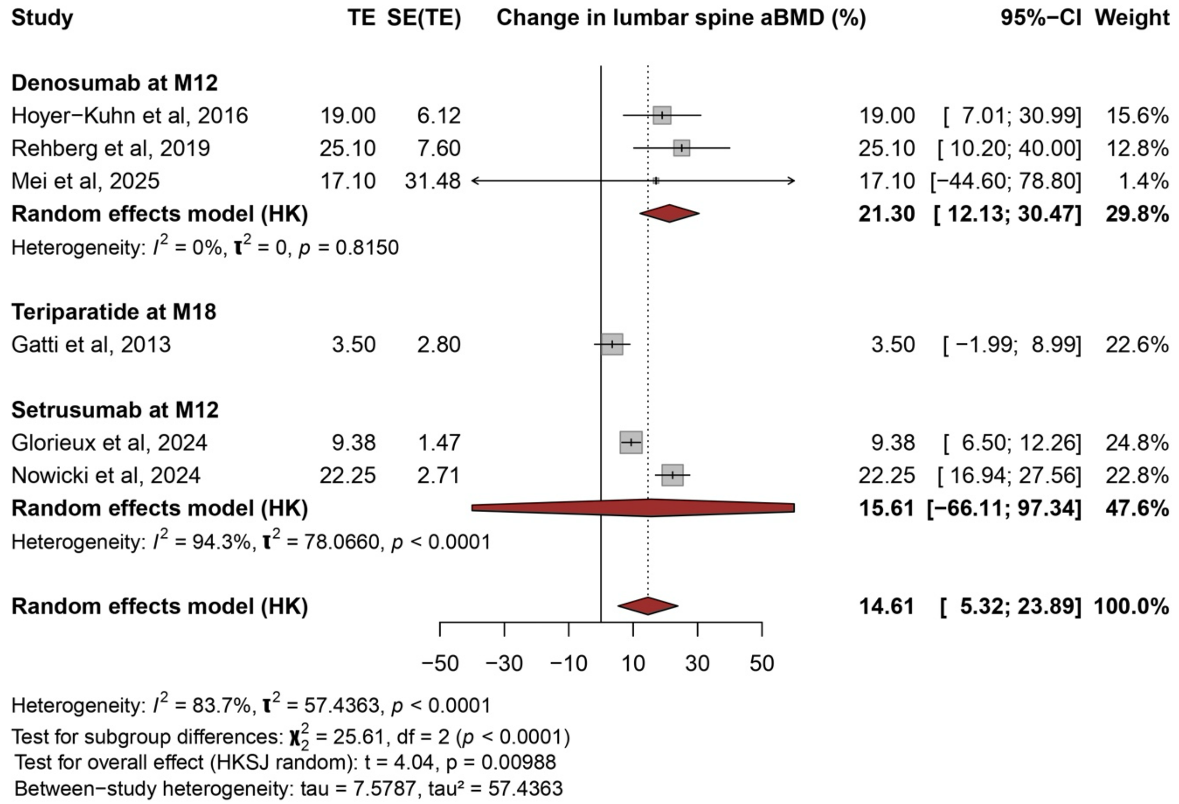

Note: In Mei et al., pediatric and adult cohorts were combined within the trial, and only the combined effect was included as a single entry in the primary meta-analysis to avoid double counting.

Figure S1b Pediatric subgroup meta-analysis of percentage change from baseline in lumbar spine aBMD in pediatric patients with osteogenesis imperfecta treated with denosumab (12 months) or setrusumab (12 months), restricted to non-randomized evidence (single-arm and/or self-controlled studies; no RCTs included) [29,34,38,40]. Random-effects forest plot using the Hartung–Knapp–Sidik–Jonkman method shows study-level estimates and pooled effects for each agent, indicating that both treatments were associated with increases in lumbar spine aBMD at 12 months (denosumab: 21.72%, 95% CI 10.96% to 32.47%; setrusumab: 22.12%, 95% CI 18.85% to 25.39%), with minimal within-agent heterogeneity (denosumab: I^2 =0%; setrusumab: single study). There was no evidence of a difference between agents in this pediatric subgroup (test for subgroup differences p=0.88).

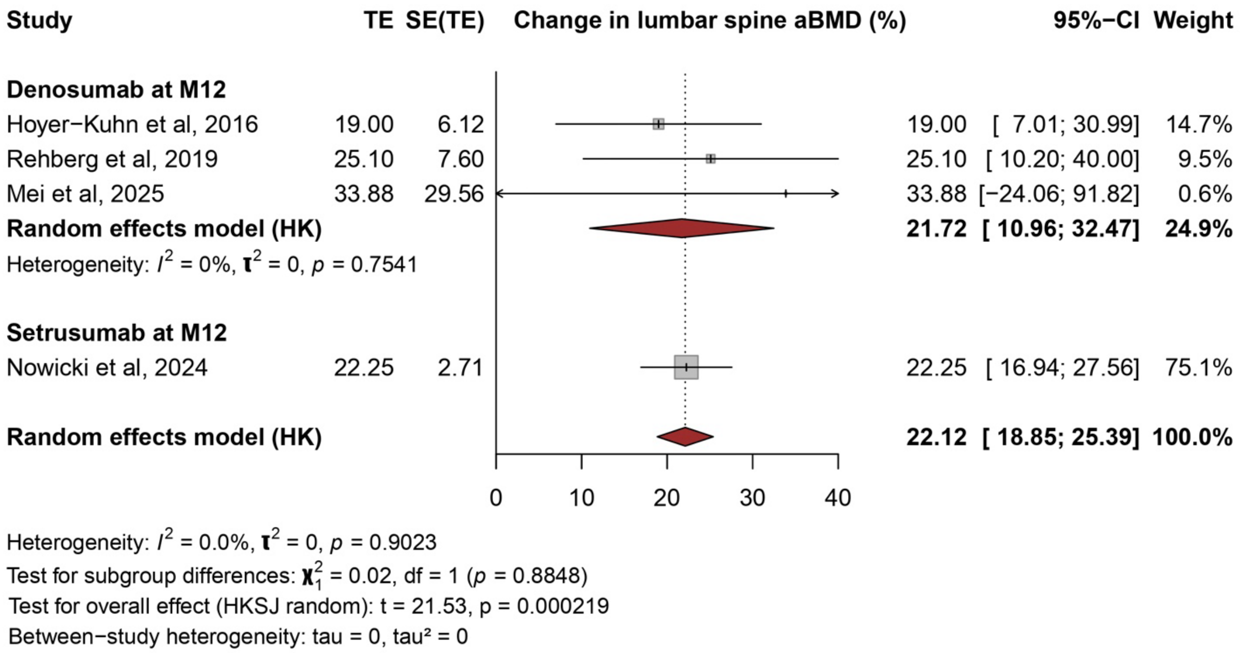

Figure S1c Adult subgroup meta-analysis of percentage change in lumbar spine aBMD in adult patients with osteogenesis imperfecta treated with denosumab (12 months), teriparatide (18 months), or setrusumab (12 months), restricted to non-randomized evidence (single-arm and/or self-controlled studies; no RCTs included) [32,33,38]. Random-effects forest plot using the Hartung–Knapp–Sidik–Jonkman method shows study-level estimates and pooled effects for each agent, indicating increases from baseline for denosumab (3.11%, 95% CI −10.53% to 16.75%), teriparatide (3.50%, 95% CI −1.99% to 8.99%), and setrusumab (9.38%, 95% CI 6.50% to 12.26%). The overall pooled estimate across agents was 6.66% (95% CI −2.38% to 15.71%), with moderate heterogeneity, ($I^2 = 49.4\%$). There was no evidence of a difference between agents in this adult subgroup (test for subgroup differences p=0.14).

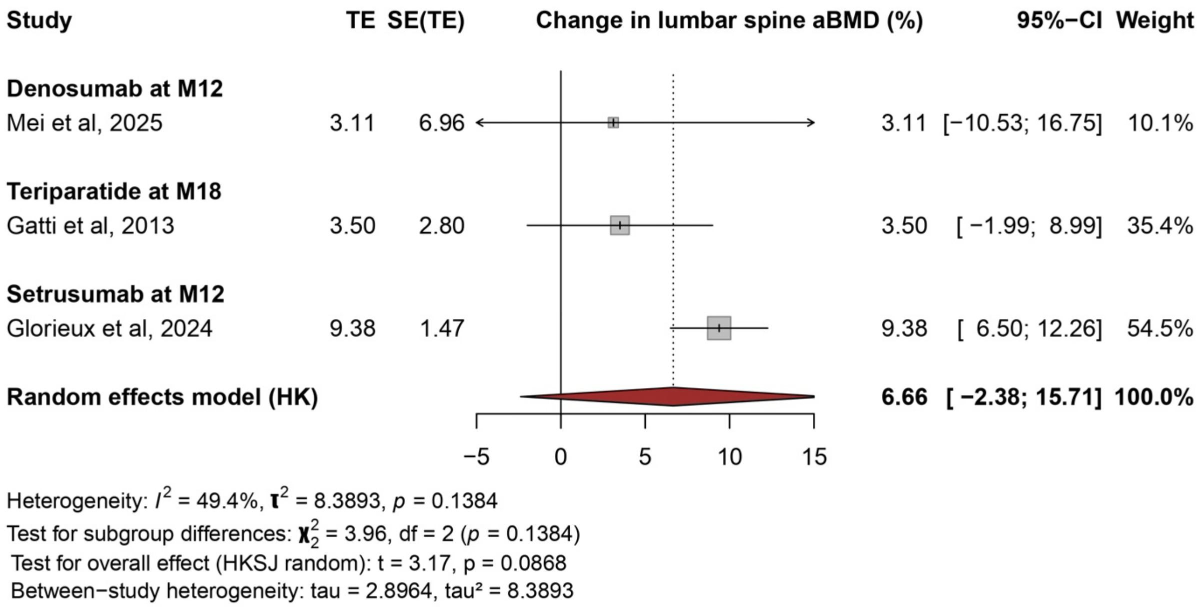

Figure S1d Pooled percentage change from baseline in lumbar spine areal bone mineral density (aBMD) following biologic therapy in patients with osteogenesis imperfecta, restricted to randomized controlled evidence (RCTs only) [36,37,39]. Forest plot from a random-effects meta-analysis using the Hartung–Knapp–Sidik–Jonkman method, showing study-level estimates and pooled effects with 95% confidence intervals for denosumab at 12 months and teriparatide at 18 months. Overall, the pooled random-effects estimate was not statistically significant and was highly heterogeneous.

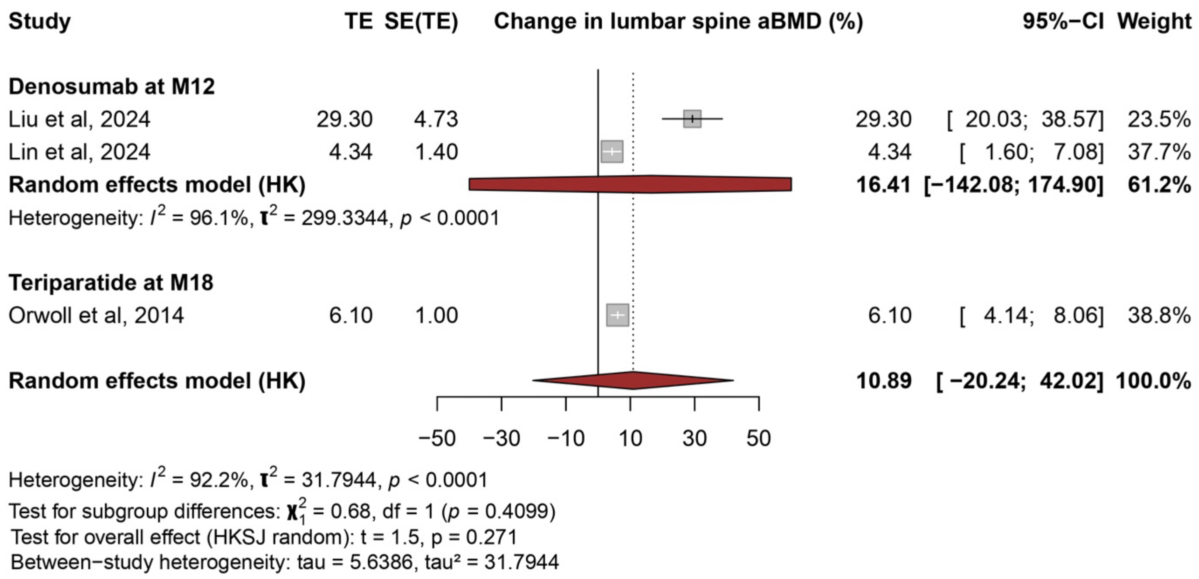

Note: In Mei et al., pediatric and adult cohorts were combined within the trial, and only the combined effect was included as a single entry in the primary meta-analysis to avoid double counting.

Figure S1e Adult subgroup meta-analysis of percentage change in lumbar spine aBMD in adult patients with osteogenesis imperfecta, restricted to randomized controlled evidence (RCTs only) [36,39]. Random-effects forest plot using the Hartung–Knapp–Sidik–Jonkman method shows study-level estimates and pooled effects for each agent, indicating increases from baseline for denosumab (4.34%, 95% CI 1.60% to 7.08%) and teriparatide (6.10%, 95% CI 4.14% to 8.06%). The overall pooled estimate across agents was 5.49% (95% CI −5.14% to 16.12%), with moderate heterogeneity, ($I^2 = 4.4\%$). There was no evidence of a difference between agents in this adult subgroup (test for subgroup differences p=0.31).

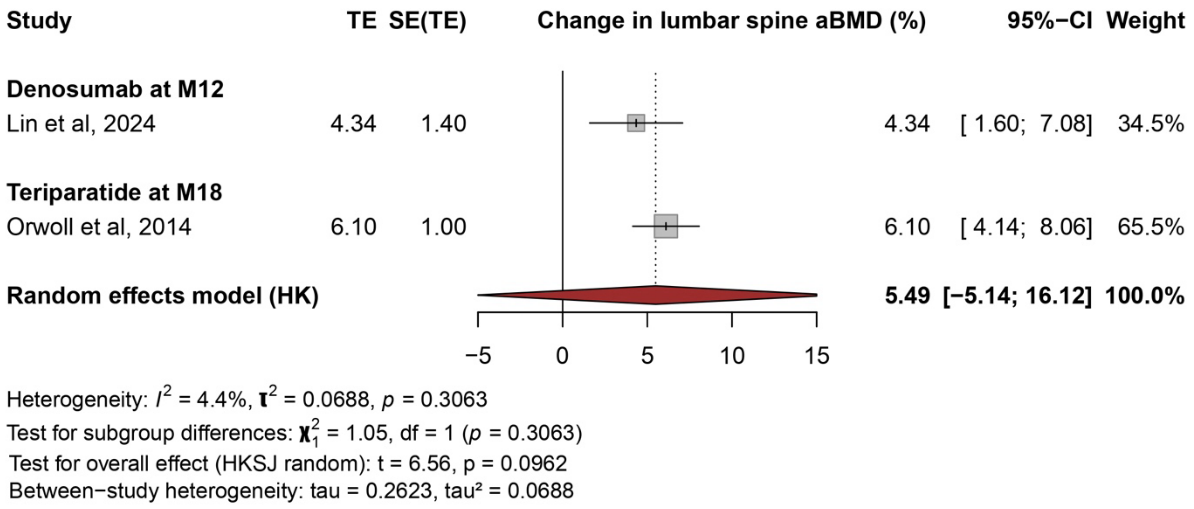

In the pediatric subgroup, only Liu's trial including children with osteogenesis imperfecta reported percentage change in lumbar spine aBMD; therefore, no meta-analysis was performed. In that trial, treatment with denosumab resulted in a mean (SD) increase of 29.3% (SD = 4.73) from baseline over a follow-up of 12 months.

Figure S2: Comparison of GPT-4o screening time for full-text versus title/abstract documents in the OI systematic review, showing significantly longer processing time for full-text screening

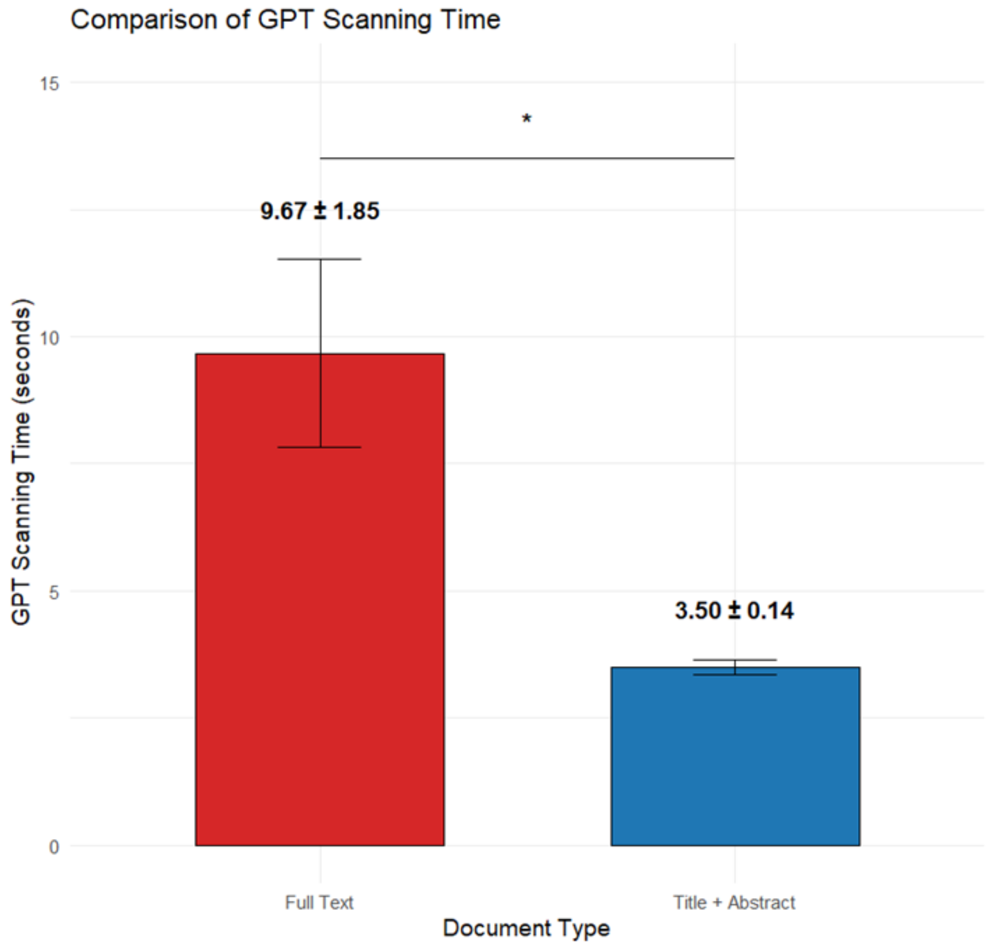

Figure S3: Risk-of-bias assessment of trials included in the meta-analysis using an adapted Cochrane Risk of Bias 2 (RoB 2) tool, showing domain-specific and overall judgments and indicating that most trials were at high risk of bias. Included studies were references [29,30,32-34,36-40].

Intention-to-treat	Unique ID	Study ID	Experimental	Comparator	Outcome	Weight	D1	D2	D3	D4	D5	Overall
	1	Liu et al. 2024	DEN	ZOL	aBMD; aBMD Z-score	42	!	+	+	-	+	-
	2	Hoyer-Kuhn et al. 2016	DEN	NA	aBMD; aBMD Z score	10	+	+	+	+	+	+
	3	Lin et al. 2024	DEN	ZOL	aBMD	25	!	+	+	-	+	-
	4	Amgen Inc. 2022	DEN	NA	aBMD Z-score	45	+	+	+	+	+	+
	5	Rehberg et al. 2019	DEN	NA	aBMD	8	+	+	+	+	+	+
	6	Mei et al. 2025	DEN	ALN	aBMD	14	-	+	+	-	!	-
	7	Owroll et al. 2014	TPTD	PBO	aBMD	29	!	+	-	+	+	-
	8	Gatti et al. 2013	TPTD	NA	aBMD	13	-	+	+	-	!	-
	9	Glorieux et al. 2024	SET	PBO	aBMD	15	+	+	+	+	+	+
	10	Nowicki et al. 2025	SET	PBO	aBMD; aBMD Z-score	14	+	+	+	+	!	!

\+ Low risk
! Some concerns
\- High risk

D1 Randomisation process
D2 Deviations from the intended interventions
D3 Missing outcome data
D4 Measurement of the outcome
D5 Selection of the reported result

Abbreviations: Dmab, denosumab; ZOL, zoledronic acid; ALN, alendronate; PBO, Placebo; TPTD, teriparatide; SET, setrusumab.

➢ Supplementary Tables

Table S3 Quality assessment results of human and GPT-based tools.

	Study	Liu et al [37]		Hoyer-Kuhn et al [34]		Lin et al [36]		Amgen Inc [30]		Rehberg et al [40]		Mei et al [38]		Orwoll et al [39]		Gatti et al [32]		Glorieux et al [33]		Nowicki et al [29]	
Five Domains	Questions	AI	Human	AI	Human	AI	Human	AI	Human	AI	Human	AI	Human	AI	Human	AI	Human	AI	Human	AI	Human
	1.1	Y	Y	Y	Y	Y	Y	Y	Y	Y	Y	NI	N	Y	Y	Y	Y	Y	Y	Y	Y
Randomization	1.2	Y	NI	PY	Y	Y	NI	Y	Y	Y	Y	NI	NI	NI	NI	PY	PN	Y	Y	Y	PY
	1.3	N	N	Y	N	N	N	Y	N	Y	N	Y	Y	N	N	Y	Y	N	N	NI	NI
	2.1	Y	Y	Y	Y	Y	Y	Y	Y	Y	Y	Y	PY	N	N	Y	Y	Y	N	N	N
	2.2	Y	Y	Y	Y	Y	Y	Y	Y	Y	Y	Y	PY	N	N	Y	Y	Y	N	N	N
	2.3	N	N	N	N	N	N	N	N	N	N	NI	N	NA	NA	N	N	NA	NA	NA	NA
Assignment*	2.4	NA	NA	NA	NA	NA	NA	NA	NA	NA	NA	NA	NA	NA	NA	NA	NA	NA	NA	NA	NA
	2.5	NA	NA	NA	NA	NA	NA	NA	NA	NA	NA	NA	NA	NA	NA	NA	NA	NA	NA	NA	NA
	2.6	Y	Y	Y	Y	Y	Y	Y	Y	Y	Y	PY	Y	Y	Y	Y	Y	Y	Y	Y	Y
	2.7	NA	NA	NA	NA	NA	NA	NA	NA	NA	NA	NA	NA	NA	NA	NA	NA	NA	NA	NA	NA
	3.1	Y	PY	Y	PN	Y	Y	Y	N	Y	Y	Y	Y	N	N	Y	Y	Y	Y	Y	PY
Missing outcome*	3.2	NA	NA	NA	PY	NA	NA	NA	N	NA	NA	NA	NA	N	PN	NA	NA	NA	NA	NA	NA
	3.3	NA	NA	NA	NA	NA	NA	NA	N	NA	NA	NA	NA	NI	PY	NA	NA	NA	NA	NA	NA
	3.4	NA	NA	NA	NA	NA	NA	NA	NA	NA	NA	NA	NA	NI	PY	NA	NA	NA	NA	NA	NA
	4.1	N	N	N	N	N	N	N	N	N	N	N	N	N	N	N	N	N	N	N	N
	4.2	N	N	N	N	N	N	N	N	N	N	N	PN	N	N	N	N	N	N	N	N
Measurement*	4.3	Y	Y	Y	Y	Y	Y	Y	Y	Y	Y	Y	PY	Y	N	Y	Y	Y	N	N	N
	4.4	Y	PY	PN	PN	N	PY	N	PN	N	PN	PY	PY	PY	NA	N	PY	Y	NA	NA	NA
	4.5	N	PY	NA	NA	NA	PY	NA	NA	NA	NA	PY	PY	PN	NA	N	PY	PN	NA	NA	NA
	5.1	Y	Y	PY	Y	NI	Y	Y	Y	NI	Y	NI	NI	NI	PY	NI	NI	Y	Y	NI	NI
Selection *	5.2	N	N	N	N	N	N	N	N	N	N	NI	PN	N	N	N	PN	N	N	NI	NI
	5.3	N	N	N	N	N	N	N	N	N	N	NI	PN	N	N	N	PN	N	N	NI	NI
Overall study risk of bias		Some	High	Low	Low	Some	High	Low	Low	Some	Low	High	High	High	High	Some	High	Some	Low	Some	Some
**Cohen's weighted κ (95%CI)**		0.779 (0.562, 0.996)		0.662 (0.357, 0.967)		0.765 (0.519, 1.011)		0.702 (0.374. 1.030)		0.763 (0.483, 1.043)		0.558 (0.357, 0.759)		0.692 (0.399, 0.986)		0.677 (0.419, 0.935)		0.562 (0.171, 0.953)		0.912 (0.805, 1.019)	
**Weighted overall κ (95%CI)**																				**0.778 (0.710, 0.846)**	

Note*: Assignment: effect of assignment to intervention; Missing data: missing outcome data; Measurement: measurement of the outcome; Selection: selection of the reported result.

Table S4: Efficacy of Included Studies

Innovative Drug	Author/ Sponsor	Intervention/ Control Group	Outcomes (Efficacy)	Key Results
Denosumab	Liu et al [37]	I: Denosumab (Subcutaneous injection every 6 months)<br>30 mg for children under 5 years old.<br>60 mg for children over 5 years old.<br>C: ZOL (Intravenous infusion once per year)<br>2.5 mg for children ≤25 kg.<br>5 mg for children >25 kg.<br>All patients received weight-adjusted oral Ca and vitamin D supplementation:<br>Ca 600 mg/day and 25OHD 125 IU/day supplementation.<br>Calcitriol (0.25 µg) administered on alternate days.	Primary:<br>aBMD (LS, TH, FN) at baseline and 6, 12 months<br>Secondary:<br>-BTMs: β-CTX, ALP, Ca, P, PTH, and 25OHD.<br>-Spinal morphometry:<br>Vertebral height and projection area.<br>Vertebral compression fracture (VCF) incidence.<br>-New fracture incidence.	1.aBMD:<br>-Significant increases at 12 months:<br>Denosumab group: LS: +29.3%; FN: +27.8%; TH: +30.2%.<br>ZOL group: LS: +32.2%.; FN: +47.1%.; TH: +41.1%.<br>FN and TH aBMD increases were significantly higher in the ZOL group than the denosumab group ($p < 0.05$).<br>2. BTMs:<br>-β-CTX:<br>Denosumab group: No significant change.<br>ZOL group: Significant decrease ($p < 0.001$).<br>-ALP:<br>Significant decreases in both groups ($p < 0.05$ for denosumab; $p < 0.001$ for ZOL).<br>-Ca:<br>Denosumab group: Significant increase ($p < 0.001$).<br>ZOL group: No significant change.<br>-PTH:<br>Denosumab group: decrease<br>ZOL group: No significant change.<br>-Spinal morphometry:<br>Vertebral height and projection area: Significant increases in both groups ($p < 0.001$). No significant

Innovative Drug	Author/ Sponsor	Intervention/ Control Group	Outcomes (Efficacy)	Key Results
				differences in morphometry changes between groups.<br>3. New fractures:<br>Denosumab group: 9 patients (21.4%).<br>ZOL group: 8 patients (19.0%).<br>No significant differences between groups.
Denosumab	Hoyer-Kuhn et al [34]	I: Denosumab (Subcutaneous injection of 1 mg/kg body weight every 12 weeks for 48 weeks)<br>C: NA<br>All patients received weight-adjusted oral calcium (Ca) and vitamin D supplementation:<br><15 kg: 250 mg Ca twice daily (0-14 days post-injection), 250 mg Ca once daily (15-28 days post-injection), and 500 IU vitamin D daily (0-28 days)<br>15-30 kg: 500 mg Ca twice daily (0-14 days post-injection), 500 mg Ca once daily (15-28 days post-injection), and 500 IU vitamin D daily (0-28 days)<br>30 kg: 1000 mg Ca twice daily (0-14 days post-injection), 1000 mg Ca once daily (15-28 days post-injection), and 1000 IU vitamin D daily (0-28 days)	Primary:<br>aBMD (LS, L2-L4) at 12 months<br>Secondary:<br>-Bone metabolism markers: DPD/crea, NTX, Osteocalcin, PTH, Ca, 25OHD<br>-Spine morphometry:<br>Anterior-posterior index and concavity index of lumbar vertebrae (L2-L4).<br>Koerber morphometry score for vertebral deformities.<br>-Mobility:<br>Gross Motor Function Measurement (GMFM-88).<br>One-minute and six-minute walking tests.	1. aBMD:<br>- aBMD (LS, L2-L4) at 12 months:<br>Absolute values: 0.507 ± 0.187 g/cm² to 0.612 ± 0.229 g/cm² ($p < 0.001$).<br>-Z-scores: −2.23 ± 2.03 to −1.27 ± 2.37 ($p = 0.0006$).<br>-Mean relative change in aBMD: +19% (95% CI: 7%-31%).<br>-Significant increase in lumbar vertebral height: 1.94 ± 0.35 cm to 2.0 ± 0.38 cm ($p = 0.03$).<br>2. Bone metabolism markers:<br>-DPD/crea: Decreased within 8 days after each injection.<br>-Serum Ca: Decreased post-injection but returned to baseline by the next visit.<br>-NTX: Increased over the trial period.<br>-Osteocalcin and PTH: Decreased over the trial period.<br>-Vitamin D: Insufficiency (10-20 µg/L) observed in 14/60 analyses, deficiency (<10 µg/L) observed in 1 case.<br>3. Spine morphometry:

Innovative Drug	Author/ Sponsor	Intervention/ Control Group	Outcomes (Efficacy)	Key Results
				-No significant changes in anterior-posterior index (p = 0.30) or concavity index (p = 0.92).<br>-Koerber morphometry score: Mean change +1.5 points (p = 0.63).<br>4. Mobility:<br>-GMFM-88: Increased by 2.72% (77.58 ± 31.64% to 80.30 ± 31.06%, p = 0.16).<br>-One-minute walking test: Increased by 12.7% (86.6 ± 26.8 m to 97.6 ± 18.0 m, p = 0.14).<br>-Six-minute walking test: Increased by 10.01% (486.5 ± 166.5 m to 535.2 ± 159.8 m, p = 0.06).
Denosumab	Lin et al [36]	I: Denosumab (60 mg subcutaneous injection every 6 months for 12 months)<br>C: Zoledronic acid (5 mg intravenous infusion once for 12 months)<br>All patients were supplemented with 600 mg of calcium and 0.25 μg of calcitriol daily.	Primary:<br>aBMD (LS; FH; TH) at baseline, 6 and 12 months of treatment<br>Secondary:<br>-Trabecular bone score (TBS)<br>-Bone turnover biomarkers (BTMs): Serum β-CTX, ALP<br>-Biochemical parameters: Serum Ca, P, PTH, and 25OHD<br>-New fractures	1.aBMD:<br>-aBMD at the LS:<br>Denosumab: +4.34% (p = 0.005).<br>Zoledronic acid: +4.92% (p = 0.006).<br>-aBMD at the TH:<br>Denosumab: +1.45% (p = 0.023).<br>Zoledronic acid: +2.02% (p = 0.016).<br>-No significant changes in FN aBMD for either group.<br>2.TBS:<br>Denosumab: +1.39%.<br>Zoledronic acid: +2.70%.<br>No significant differences in TBS changes between groups.<br>3.BTMs:<br>Serum β-CTX:

Innovative Drug	Author/ Sponsor	Intervention/ Control Group	Outcomes (Efficacy)	Key Results
				Denosumab: −49.71% at 6 months and −45.64% at 12 months ($p < 0.05$ vs baseline).<br>Zoledronic acid: −59.88% at 6 months and −49.18% at 12 months ($p < 0.05$ vs baseline).<br>ALP: similar reductions in both groups with no significant differences.<br>4.Biochemical parameters: similar reductions for Ca, P, PTH, and 25OHD in both groups with no significant differences.<br>5. Fracture incidence:<br>Denosumab: 2 new fractures (8.0%).<br>Zoledronic acid: 1 new fracture (3.8%).<br>No significant differences between groups (p = 0.610).
Denosumab	Amgen Inc [30]	I: Denosumab (Subcutaneous injection: 1 mg/kg (maximum 60 mg) every 6 months for 36 months)<br>C: NA<br>(Dosing regimen adjusted to every 3 months after early efficacy and pharmacokinetics data analysis.)<br>Supplementation:<br>Serum albumin-corrected Ca and P monitored regularly.<br>25OHD: Supplementation	Primary:<br>-aBMD Z-score (LS TH FN) at 6 and 12 months.<br>-Fracture:<br>Percentage of participants with at least 1 X-ray confirmed long bone or vertebral fracture.<br>Percentage of participants with improving vertebral fractures.<br>Secondary:<br>-Growth outcomes:<br>Change in growth velocity (height-for-age, weight-for-age,	1.BMD:<br>-LS BMD Z-score:<br>Significant increase at 12 months: +1.009 ± 0.119 ($p < 0.05$).<br>Increase at 6 months: +0.925 ± 0.078.<br>-Proximal femur BMD Z-scores:<br>Total hip at 12 months: +0.799 ± 0.082.<br>Femoral neck at 12 months: +0.769 ± 0.067.<br>2. Participants with at least 1 X-ray confirmed long bone or vertebral fracture:<br>Q6M regimen: 28.3%.<br>Q3M regimen: 26.7%.<br>-Participants with improving vertebral fractures

Innovative Drug	Author/ Sponsor	Intervention/ Control Group	Outcomes (Efficacy)	Key Results
		administered if baseline level <20 ng/mL.	BMI-for-age Z-scores).<br>-Patient-reported outcomes: Change in Child Health Questionnaire-Parent Form (CHQ-PF-50) physical and psychological summary scores.<br>-Change in Childhood Health Assessment Questionnaire (CHAQ) disability index score.<br>-Change in Wong-Baker Faces Pain Rating Scale (WBFPRS).<br>-Serum concentrations of denosumab and bone turnover markers:<br>CTX-I and BSAP.	during Q3M dosing: 27.7%.<br>3. Growth velocity Z-scores showed no significant changes at 12 months:<br>Height-for-age: −0.01 ± 0.43.<br>Weight-for-age: +0.01 ± 0.53.<br>BMI-for-age: −0.07 ± 0.52.<br>4. Patient-reported outcomes:<br>-CHQ-PF-50:<br>Physical summary score: −0.98 ± 15.41.<br>Psychological summary score: +0.85 ± 8.57.<br>-CHAQ disability index score: −0.06 ± 0.46.<br>-WBFPRS: No change (0.0 ± 1.7).<br>5. Bone turnover markers:<br>CTX-I: Decreased significantly at multiple time points ($p < 0.05$).<br>BSAP: Decreased significantly ($p < 0.05$).
Denosumab	Rehberg et al. [40]	I: Denosumab 1 mg/kg every 12 weeks for 36 weeks.<br>C: Prior bisphosphonate period. Calcium and vitamin D supplementation after denosumab injection.	Primary:<br>-LS aBMD<br>Secondary:<br>-Trabecular bone score (TBS)<br>-Percentage change in aBMD and [illegible]<br>-Height/weight Z-scores	1. aBMD:<br>Bisphosphonate: +6.2% ± 8.4%<br>Denosumab: +25.1% ± 7.6%; P=0.007<br><br>2. TBS:<br>Bisphosphonate: +2.1% ± 5.0%<br>Denosumab: +6.7% ± 4.1%; P=0.132<br><br>3. aBMD vs TBS during denosumab:<br>aBMD +25.1% vs TBS +6.7%; P<.01

Innovative Drug	Author/ Sponsor	Intervention/ Control Group	Outcomes (Efficacy)	Key Results
				4. Individual response:<br>Denosumab: increase in aBMD and TBS in all 8 patients<br>Bisphosphonate: increase in aBMD and TBS in 6/8 patients<br><br>5. Height/weight:<br>Increased during study; Z-score changes NS
Denosumab	Mei et al [38]	I: Denosumab (Subcutaneous injection).<br>Pediatric: 1 mg/kg every 3 months (≤60 mg/dose).<br>Adult: 60 mg every 6 months.<br>C: Alendronate (Oral). 70 mg/week.<br>All participants received calcium 300–600 mg/day and vitamin D ≥400 IU/day.	Primary:<br>-aBMD (LS and FN) change at baseline and 12 months.<br>-New fracture incidence.<br>Secondary:<br>-Bone turnover markers (CTX, OC, ALP, Ca, P, PTH, 25OHD).<br>-Vertebral area loss.<br>-Height velocity.	1. aBMD:<br>No significant intergroup differences in BMD change.<br>-LS<br>Pediatric:<br>Denosumab: +30.3% ($p<0.001$)<br>Alendronate: +24.0% ($p<0.001$)<br>Adult:<br>Denosumab: +2.6% ($p=0.100$)<br>Alendronate: +5.1% ($p=0.010$)<br>-FN<br>Pediatric:<br>Denosumab: +38.7% ($p=0.001$).<br>Alendronate: +15.7% ($p<0.001$).<br>Adult:

Innovative Drug	Author/ Sponsor	Intervention/ Control Group	Outcomes (Efficacy)	Key Results
				Denosumab: +4.4% (p=0.051).<br>Alendronate: +1.6% (p=0.051).<br>2. BTMs:<br>Pediatric: Denosumab transient CTX suppression with rebound by month 9. Persistent OC suppression.<br>Adult: Denosumab sustained CTX reduction (49% at 12 months, p<0.05). Alendronate sustained CTX and OC reductions in both age groups.<br>3. Vertebral morphometry:<br>Pediatric: Denosumab vertebral area loss reduced significantly (−14.6%, p=0.029). Two cases with fracture remodeling. Alendronate reduction not significant (−8.8%, p=0.296).<br>4. Height velocity: Pediatric Denosumab +5.8% vs Alendronate +2.5% (p=0.004).<br>5. New fractures: Pediatric Denosumab 2 cases (0.83/year). Pediatric Alendronate similar rate (0.25/year, ns). Adults none in either group.
Teriparatide	Orwoll et al [39]	I: Teriparatide (20 μg subcutaneous daily for 18 months.)<br>C: Placebo (subcutaneous injection daily for 18 months.)<br>All participants received daily	Primary:<br>aBMD (LS, TH FN) at baseline, 6 ,12,18 months.<br>Secondary:<br>-Bone remodeling markers at baseline, 6, 12, and 18 months:	1. aBMD:<br>-LS: Significant difference ($p < 0.05$)<br>Teriparatide group: +6.1%.<br>Placebo group: +2.8%.<br>-TH: Significant difference ($p < 0.001$)<br>Teriparatide group: +2.6%.

Innovative Drug	Author/ Sponsor	Intervention/ Control Group	Outcomes (Efficacy)	Key Results
		calcium (1,000 mg) and vitamin D (800 IU) supplements.	P1NP, NTX<br>-Vertebral vBMD at 18 months<br>-Estimated vertebral strength at 18. months.<br>-Fracture incidence: Self-reported fractures at each visit.	Placebo group: -2.4%.<br>-FN:<br>Teriparatide group: Significant increase (+3.7%, $p < 0.05$).<br>Placebo group: No significant change.<br>2. Bone remodeling markers:<br>P1NP: Increased significantly in the teriparatide group (+135%, $p < 0.001$).<br>NTX: Increased significantly in the teriparatide group (+64%, $p < 0.001$).<br>3. Vertebral vBMD: Significant difference ($p < 0.05$)<br>Teriparatide group: +18%.<br>Placebo group: -5%.<br>4. Estimated vertebral strength: Significant difference ($p < 0.05$)<br>Teriparatide group: +15%.<br>Placebo group: -2%.<br>5. Fracture incidence:<br>Teriparatide group: 11/38 (29%).<br>Placebo group: 14/40 (36%).<br>No significant difference between groups.
Teriparatide	Leali et al [35]	I: Teriparatide (20 µg subcutaneously daily for 2 years)<br>C: Neridronate (Intravenous infusion, 100 mg every 3 months for 2 years.)<br>Both groups received supplemental	Primary:<br>aBMD (LS, FN) at baseline and 24 months.<br>Secondary:<br>-BTMs at baseline and 6, 12, 24 months:	1. LS BMD at 24 months<br>Teriparatide: Significant increase (+5.1%, $p < 0.001$).<br>Neridronate: Decrease (-1.6%, $p < 0.001$).<br>2. Bone turnover markers (BTMs):<br>-Teriparatide group:

Innovative Drug	Author/ Sponsor	Intervention/ Control Group	Outcomes (Efficacy)	Key Results
		vitamin D (600 IU/day) and calcium (1,200 mg/day).	Bone formation markers: BSAP, PICP<br>Bone resorption markers: DPD, NTX assessed at baseline, 6, 12, and 24 months.<br>-New fracture incidence at 6 months<br>-Pain: VAS for bone pain ssessed at baseline, 6, 12, 18, and 24 months.<br>-Quality of life:<br>Short Form-8 health survey, including physical and mental health domains	Bone formation markers: Significant increases at all time points (PICP +236.2%, BSAP +60.9%; $p < 0.001$).<br>Bone resorption markers: Significant decreases at all time points (DPD -229.2%, NTX -28.9%; $p < 0.001$).<br>-Neridronate group:<br>Bone formation markers: Modest increases (PICP +178.7%, BSAP +37.8%; $p > 0.05$).<br>Bone resorption markers: Modest decreases (DPD -272.8%, NTX -46.8%; $p > 0.05$).<br>3. New fractures:<br>Teriparatide: 8 patients (16.33%).<br>Neridronate: 13 patients (26.53%).<br>Difference not statistically significant ($p = 0.10$).<br>4. Pain:<br>Significant improvement in VAS scores in both groups at all time points ($p < 0.001$).<br>Similar reductions in mean VAS scores between groups.<br>5. Quality of life:<br>Significant improvements in both groups: Better outcomes in the teriparatide group for the physical component summary (+4.42) and mental health domain (+2.43).

Innovative Drug	Author/ Sponsor	Intervention/ Control Group	Outcomes (Efficacy)	Key Results
Teriparatide	Gatti et al [32]	I:Teriparatide (daily subcutaneous injection for 18 months) ；<br>C: NA<br>Calcium intake maintained above 1,000 mg/day through diet or supplementation.<br>Vitamin D3 (25,000 IU monthly) supplementation when serum 25(OH) vitamin D levels fell below 20 ng/mL.	Primary:<br>aBMD (LS, FN) at baseline and every 6 months for 18 months<br>Secondary:<br>-BTMs: P1NP，bAP，CTX<br>-Wnt pathway inhibitors: Serum DKK1, Serum sclerostin	1.BMD:<br>Lumbar spine BMD: Significant increase of 3.5% ($p = 0.001$) after 18 months.<br>Hip BMD: No significant changes observed.<br>2.Bone turnover markers:<br>Significant increases in P1NP, bAP, and CTX, peaking at 12 months,suggests a strong osteoblastic response to teriparatide.<br>P1NP: Increased fourfold.<br>bAP: Increased twofold.<br>3.Wnt pathway inhibitors:<br>Serum DKK1: Significant gradual increase, observed as early as 6 months ($p < 0.01$).<br>Serum sclerostin: Nonsignificant increase.
Fresolimumab	Song et al [14]	I: Fresolimumab (intravenous infusion)<br>1 mg/kg ($n = 4$)<br>4 mg/kg ($n = 4$)<br>C: NA	Primary:<br>LS aBMD at baseline, 3 and 6 months<br>Secondary:<br>-Bone remodeling markers: Ocn, CTX, P1NP.	1. Bone remodeling markers:<br>-Ocn: Significant decrease in the 4 mg/kg group compared to 1 mg/kg ($p = 0.0045$).<br>-CTX and P1NP: No significant differences between dose groups.<br>2. LS aBMD<br>-1 mg/kg group:<br>Two participants with OI type IV showed robust increases (6.8% and 8.6%).<br>Participant with OI type VIII showed no change.<br>-4 mg/kg group:<br>One participant had a 7.6% increase.<br>Two participants had smaller increases (2.9% and

Innovative Drug	Author/ Sponsor	Intervention/ Control Group	Outcomes (Efficacy)	Key Results
				1.3%).<br>Two participants with OI type III had decreases in aBMD due to scoliosis (measurement error) and immobility (fracture).
Romosozumab	El-Maouche et al [31]	I: Romosozumab (subcutaneous injection).<br>Each age group received three monthly doses at one of three dose levels: 1 mg/kg, 3 mg/kg, or 5 mg/kg (four patients per cohort).<br>C: NA	Primary:<br>Pharmacokinetic parameters.<br>Secondary:<br>-BTMs (serum type 1 collagen C-telopeptide, procollagen type 1 N-terminal propeptide)<br>-LS aBMD change from baseline	1. Pharmacokinetics: Systemic exposure increased with dose level in both age groups, higher in adolescents than children.<br>2. Bone turnover markers: No clear dose response in serum type 1 collagen C-telopeptide levels. P1NP increased to 58.6% in adolescents and 28.4% in children at day 15 after dosing, returning towards baseline before next dose.<br>3. LS aBMD: Mean percentage increase from baseline to day 169 after 3 months ranged from 7.1% to 15.0% in adolescents and 7.1% to 12.7% in children.
Setrusumab	Glorieux et al [33]	I: Setrusumab (administered in three doses (2 mg/kg, 8 mg/kg, and 20 mg/kg) via monthly intravenous infusion for 12 months.)<br>C: Placebo group (initially randomized participants were reassigned to receive setrusumab 20 mg/kg open label after a median of 5 months).<br>All participants received concomitant	Primary:<br>-Radial trabecular vBMD at baseline and month12<br>Secondary:<br>-Radial bone strength: failure load and stiffness<br>-Changes in total and cortical vBMD of the radius and tibia<br>-aBMD (LS, FN, TH, Total body)	1. Change in radial trabecular vBMD at 12 months did not reach statistical significance<br>2. Statistically significant increase in bone strength in the 20 mg/kg group (p=.006 for failure load, p=0.011 for stiffness)<br>3. aBMD:<br>LS:<br>20 mg/kg group: +9.0% (p < .001).<br>8 mg/kg group: +6.4% (p < .001).<br>2 mg/kg group: +3.2% (p = .035).

Innovative Drug	Author/ Sponsor	Intervention/ Control Group	Outcomes (Efficacy)	Key Results
		therapy with active vitamin D and/or calcium supplements.	-BTMs: P1NP, Ocn, BSAP, and CTX<br>-Fracture rates	TH:<br>20 mg/kg group: +5.2% (p = .004).<br>8 mg/kg group: +3.9% (p = .003).<br>2 mg/kg group: +2.2% (p = .027).<br>FN:<br>Significant improvements in the 8 mg/kg (p = .038) and 20 mg/kg (p = .012) groups.<br>Total Body aBMD<br>Significant increases in the 8 mg/kg (p = .026) and 20 mg/kg (p = .005) groups.<br>4. Bone formation markers (P1NP, OC, BSAP) increased significantly after treatment initiation in the 8 mg/kg and 20 mg/kg groups, peaking at 1–3 months and tapering towards baseline at 12 months. Bone resorption marker CTx1 decreased initially and returned to baseline at 12 months.<br>5. Significant improvements in cortical and total vBMD of the tibia in the 20 mg/kg group (p < 0.001 and p = 0.02, respectively).<br>6. Fracture Rates<br>20 mg/kg group: 0.19 fractures/participant/year (lowest among all groups)
Setrusumab	Nowicki et al [29]	I: Setrusumab (intravenous infusion).<br>C:NA	Primary:<br>-LS BMD percentage change from baseline at Month 6 and Month 12.	1. LS BMD: Mean change from baseline at Month 6 was +14.19% (SE 2.15%, p<0.0001). At Month 12 was +22.25% (SE 2.71%, p<0.0001).

Innovative Drug	Author/ Sponsor	Intervention/ Control Group	Outcomes (Efficacy)	Key Results
			-LS BMD Z-score change from baseline at Month 6 and Month 12. Secondary:	2. LS BMD Z-score: Mean change from baseline at Month 6 was +0.85 (SE 0.13, $p<0.0001$). At Month 12 was +1.25 (SE 0.17, $p<0.0001$).
			-Annualized fracture rate change from pre-treatment to treatment period.	3. Fracture rate: Median annualized pre-treatment fracture rate was 0.72. After a mean of 16 months of treatment median annualized fracture rate was 0. Calculated fracture rate reduction was 67% ($p=0.0014$).

Abbreviations: aBMD, areal bone mineral density; vBMD, volumetric bone mineral density; LS, lumbar spine; TH, total hip; FN, femoral neck; TBS, trabecular bone score; BTMs, bone turnover markers; BSAP, bone-specific alkaline phosphatase; PICP, carboxy-terminal extension peptide of procollagen type I; DPD, free deoxypyridinoline; NTX, N-terminal telopeptide; Ocn, osteocalcin; CTX, C-terminal telopeptide; P1NP, N-terminal propeptide of type 1 procollagen; VAS, Visual Analogue Scale; CHQ-PF-50, Child Health Questionnaire-Parent Form 50; CHAQ, Childhood Health Assessment Questionnaire; WBFPRS, Wong-Baker Faces Pain Rating Scale; Q6M, every 6 months; Q3M, every 3 months; NA, not applicable.

S4a Qualitative Synthesis of Individual Trial (Non-pooled) Efficacy Findings

Teriparatide is the only biologic directly compared to BPs for lumbar spine aBMD in a head-to-head RCT in adults with type I OI, as it is contraindicated in children due to the risk of osteosarcoma. In the RCT by Leali et al. [35], daily subcutaneous teriparatide (20 µg) led to a 5.1% increase in aBMD at 24 months, versus a 1.6% decrease with neridronate. ($p < 0.001$). This is consistent with Gatti et al. [32], who observed a 3.5% increase after 18 months of teriparatide in neridronate-pretreated postmenopausal women with type I OI ($p = 0.001$). However, post-hoc exploratory analyses by Orwoll et al.[1] showed teriparatide had limited efficacy in more severe OI forms (types III and IV).

A similar pattern was observed with fresolimumab in adults: in a phase I trial, Song et al. [14] reported lumbar spine aBMD increases of 6.8–8.6% at 6 months patients with type IV OI, although gains with 4 mg/kg were transient and followed by a rebound decline after 3 months. No benefit was seen in more severe forms (type III or VIII), likely due to confounding factors such as scoliosis and post-fracture immobility that accelerate bone loss. Notably, the 3-month aBMD increase with 1 mg/kg fresolimumab exceeded the 2% gain at 6 months reported for teriparatide in Orwoll et al. [39], suggesting fresolimumab may offer a more rapid but possibly less durable effect in adults.

Setrusumab has been evaluated in two phase 2 trials [29, 33]. including a randomized, double-blind, dose-finding trial in adults (Asteroid[2]) and an open-label, randomized dose-finding study in children and young adults (ORBIT Phase 2[3]). In adults, Glorieux et al.[2] reported a clear dose-response, with lumbar spine aBMD increasing by 9.4% (SD = 1.5) at 12 months in the 20 mg/kg group, and significant gains also observed at 2 and 8 mg/kg groups. In contrast, in patients aged 5 to 26 years, Nowicki et al.[3] found both 20 and 40 mg/kg doses led to significant aBMD improvements at 3 and 6 months, but no additional benefit was seen with 40 mg/kg compared to 20 mg/kg. These findings suggest that setrusumab confers robust aBMD gains across age groups, with maximal gains observed at 20 mg/kg, and a possible ceiling effect at higher doses. Similar patterns were observed with romosozumab, another sclerostin antibody, in a phase 1 dose-ascending study in children and adolescents with OI showed dose-related increases in P1NP and early lumbar spine aBMD gains of approximately 7–15% after only 3 months of treatment [31]. Taken together with the pediatric ORBIT data for setrusumab, these findings suggest that sclerostin inhibition can rapidly and substantially increase spine BMD in younger OI patients with an acceptable short-term safety profile, complementing the more restricted efficacy and use of teriparatide and fresolimumab in adults.

Table S5 Safety outcomes in trials that reported safety data

**Outcomes**	Denosumab					Romosozumab	Teriparatide	Fresolimumab	Setrusumab		
	Liu et al. [37] (n=42)	Hoyer-Kuhn et al. [34] (n=10)	Lin et al. [36] (n=25)	Amgen Inc. [30] (n=153)	Mei et al. [38] (n=18)	El-Maouche et al. [31] (n=25)	Gatti et al. [32] (n=13)	Song et al. [14] (n=8)	Glorieux et al. [33] (n=111)	Nowicki et al. [29] (M6, n=24)	Nowicki et al. [29] (M12, n=24)
Any adverse events	-	76	5 (20.00)	141 (92.16)	-	12 (48.00)	-	-	104 (93.69)	-	-
Serious adverse events	10 (23.81)	-	0 (12.00)	52 (33.99)	0 (0.00)	2 (8.00)	-	-	20 (18.02)	-	0 (0.00)
Treatment-related adverse events	-	16	-	-	-	-	-	-	4 (3.60)	-	12 (50)
Withdrawal due to adverse event	-	-	-	-	-	0 (0.00)	-	-	4 (3.60)	-	-
All-cause mortality	0 (0.00)	-	-	0 (0.00)	0 (0.00)	0 (0.00)	-	-	-	-	-
**Metabolic disturbances**											
Hypocalcemia	1 (2.38)	1	0 (0.00)	15 (9.80)	2 (11.11) (1 child, 1 adult)	-	-	-	-	-	-
Hypercalcemia	13 (30.95)	-	0 (0.00)	28 (18.30)	5 (27.78) (all pediatric)	-	-	-	-	-	-
Hypercalcemia crisis	6 (14.29)	-	-	-	-	-	-	-	-	-	-
Metabolic acidosis	-	-	-	1 (0.65)	-	-	-	-	-	-	-
Hypercalciuria	-	-	-	49 (32.03)	-	-	-	-	-	-	-
**Musculoskeletal and connective tissue disorders**											
Arthralgia	-	10	-	70 (45.75)	4 (22.22) (all pediatric)	-	-	-	1 (0.90)	-	-

Muscle pain	-	3	-	-	4 (22.22) (all pediatric)	-	-	-	-	-	-
Back pain	-	-	-	50 (32.68)	-	-	-	-	-	-	-
Bone pain	9 (21.43)	-	3 (12.00)	21 (13.73)	-	-	-	-	1 (0.90)	1 (4.17)	-
Infusion-related pain	-	-	-	-	-	-	-	-	-	1 (4.17)	-
Bronchitis	-	-	-	-	-	-	-	-	-	-	-
Myalgia	-	-	0 (0.00)	8 (5.23)	-	-	-	-	-	-	-
Pain between ribs	-	1	-	-	-	-	-	-	-	-	-
Pain in extremity	-	-	-	59 (38.56)	-	-	-	-	-	-	-
Tenosynovitis	-	-	-	1 (0.65)	-	-	-	-	-	-	-
Haemarthrosis	-	-	-	1 (0.65)	-	-	-	-	-	-	-
Scoliosis	-	-	-	1 (0.65)	-	-	-	-	-	-	-
Joint abscess	-	-	-		-	-	-	-	1 (0.90)	-	-
Knee deformity	-	-	-	1 (0.65)	-	-	-	-	-	-	-
Limb deformity	-	-	-	2 (1.31)	-	-	-	-	-	-	-
Osteochondrosis	1 (2.38)	-	0 (12.00)	1 (0.65)	-	-	-	-	-	-	-
Bleeding from skinscab	-	-	-		-	-	-	1	-	-	-
**Infections and infestations**											
Gastroenteritis	-	-	-	8 (5.23)	-	-	-	-	-	-	-
Influenza	-	-	-	13 (8.50)	-	-	-	-	-	-	-
Nasopharyngitis	-	-	-	23 (15.03)	-	-	-	-	-	-	-
Upper respiratory tract infection	-	-	-	9 (5.88)	-	-	-	-	-	1 (4.17)	-
Gastroenteritis viral	-	-	-	1 (0.65)	-	-	-	-	-	-	-
Device-related infection	-	-	-	-	-	-	-	-	1 (0.90)	-	-
Soft tissue infections	-	-	0 (12.00)	-	-	-	-	-	-	-	-
Wound infection	-	-	-	-	-	-	-	-	1 (0.90)	-	-
**General disorders**											

Infusion site reaction	-	-	1 (4.00)	-	-	-	-	-	12 (10.81)	7 (29.17)	-
Fever	4 (9.52)	-	-	-	-	-	-	-	-	-	-
Nausea	-	-	-	-	-	-	7 (53.85)	1	-	-	-
Epistaxis	-	-	-	11 (7.19)	-	-	-	2	-	-	-
Pain	-	-	-	13 (8.50)	-	-	-	-	-	-	-
Malaise	-	-	-	-	-	-	-	1	-	-	-
Pyrexia	-	-	0 (0.00)	18 (11.76)	-	-	-	-	-	-	-
Chills	-	-	-	-	-	-	-	-	1 (0.90)	-	-
Visual impairment	-	-	-	-	-	-	-	-	1 (0.90)	-	-
Fatigue	-	-	1 (4.00)	-	-	-	-	-	-	-	-
Anaphylaxis	-	-	-	-	-	-	-	-	1 (0.90)	-	-
**Digestive system disorders**											
Abdominal pain	-	-	-	13 (8.50)	-	-	-	-	-	-	-
Upper abdominal pain	-	-	-	10 (6.54)	-	-	-	-	-	-	-
Gastrointestinal pain	-	-	-	1 (0.65)	-	-	-	-	-	-	-
Dental caries	-	-	-	9 (5.88)	-	-	-	-	-	-	-
Cholelithiasis	-	-	-	-	-	-	-	-	1 (0.90)	-	-
Appendicitis	-	-	-	-	-	-	-	-	1 (0.90)	-	-
Vomiting	6 (14.29)	-	-	13 (8.50)	-	-	-	-	-	-	-
Constipation	1 (2.38)	-	-	-	-	-	-	-	-	-	-
Eczema	-	-	-	82.9	-	-	-	-	-	-	-
Aphthous lesion soft palate	-	-	-	-	-	-	-	-	-	-	-
**Injury**											
Fall	-	-	-	26 (16.99)	-	-	-	-	-	-	-
Contusion	-	-	-	17 (11.11)	-	-	-	-	-	-	-
Concussion	-	-	-	1 (0.65)	-	-	-	-	-	-	-

**Other systems**											
Urinary disorder	-	-	-	-	-	-	-	1	-	-	-
Cardiac disorder	-	-	-	1 (0.65)	-	-	-	-	-	-	0 (0.00)
Nervous system disorders	-	-	0 (0.00)	22 (14.38)	-	-	-	1	3 (2.70)	3 (12.50)	-
Respiratory, thoracic and mediastinal disorders	1	-	-	13 (8.50)	-	-	-	-	1 (0.90)	-	-
Psychiatric disorders	1	-	-	-	-	-	-	-	-	-	-
Reproductive system or breast disorder	1	-	-	-	-	-	-	-	-	-	-
Vascular disorder	2	-	-	-	-	-	-	-	1 (0.90)	-	-

References

1. Orwoll Es Fau - Shapiro, J., et al., *Evaluation of teriparatide treatment in adults with osteogenesis imperfecta.* (1558-8238 (Electronic)).
2. *World Congress on Osteoporosis, Osteoarthritis and Musculoskeletal Diseases (WCO-IOF-ESCEO 2025).* (1720-8319 (Electronic)).
3. Glorieux, F.H., et al., *Setrusumab for the treatment of osteogenesis imperfecta: 12-month results from the phase 2b asteroid study.* (1523-4681 (Electronic)).
