# Supplementary material for: Artificial Intelligence for Evidence Synthesis of Emerging Biologics to Improve Skeletal Health in Osteogenesis Imperfecta: Systematic Review and Meta-Analysis
Source: J Med Internet Res. 2026 Jul 10;28:e85840. doi: 10.2196/85840 (PMC13354119; doi:10.2196/85840)
Supplement: Checklist 2 [file jmir-v28-e85840-s005.pdf]

PRISMA-S Checklist

Section/topic	#	Checklist item	Location(s) Reported
**INFORMATION SOURCES AND METHODS**			
Database name	1	Name each individual database searched, stating the platform for each.	Page 6, lines 18-19
Multi-database searching	2	If databases were searched simultaneously on a single platform, state the name of the platform, listing all of the databases searched.	Page 6, lines 20-21
Study registries	3	List any study registries searched.	Page 6, lines 13.<br>Page 6, lines 21
Online resources and browsing	4	Describe any online or print source purposefully searched or browsed (e.g., tables of contents, print conference proceedings, web sites), and how this was done.	Page 6, lines 31-32
Citation searching	5	Indicate whether cited references or citing references were examined, and describe any methods used for locating cited/citing references (e.g., browsing reference lists, using a citation index, setting up email alerts for references citing included studies).	Page 6, lines 32-33, 35
Contacts	6	Indicate whether additional studies or data were sought by contacting authors, experts, manufacturers, or others.	Page 6, lines 34-35
Other methods	7	Describe any additional information sources or search methods used.	Page 6, lines 27-30
**SEARCH STRATEGIES**			
Full search strategies	8	Include the search strategies for each database and information source, copied and pasted exactly as run.	Supplementary Appendix 1 (Table S1)
Limits and restrictions	9	Specify that no limits were used, or describe any limits or restrictions applied to a search (e.g., date or time period, language, study design) and provide justification for their use.	Page 6, lines 35-38
Search filters	10	Indicate whether published search filters were used (as originally designed or modified), and if so, cite the filter(s) used.	Page 6, lines 35-36

Prior work	11	Indicate when search strategies from other literature reviews were adapted or reused for a substantive part or all of the search, citing the previous review(s).	Page 6, lines 38-39
Updates	12	Report the methods used to update the search(es) (e.g., rerunning searches, email alerts).	Page 6, lines 36
Dates of searches	13	For each search strategy, provide the date when the last search occurred.	Supplementary Appendix 1 (Table S1)
**PEER REVIEW**			
Peer review	14	Describe any search peer review process.	Page 6, lines 39-40
**MANAGING RECORDS**			
Total Records	15	Document the total number of records identified from each database and other information sources.	Supplementary Appendix 1 (Table S1)
Deduplication	16	Describe the processes and any software used to deduplicate records from multiple database searches and other information sources.	Page 6, lines 41-42, Page 7, lines 1-2

PRISMA-S: An Extension to the PRISMA Statement for Reporting Literature Searches in Systematic Reviews
Rethlefsen ML, Kirtley S, Waffenschmidt S, Ayala AP, Moher D, Page MJ, Koffel JB, PRISMA-S Group.
Last updated February 27, 2020.
